# Octahedral [(8-quinolinyl/phenanthridinyl)amine)Ga(III)] complexes, reactivity, affinity for biomolecules, anti-cancer and computational evaluations

**DOI:** 10.1007/s11144-026-03129-6

**Published:** 2026-05-14

**Authors:** Phakamani C. Dlamini, Thato T. Medupe, Lucy W. Macharia, Karabo Serala, Sharon Prince, Gregory S. Smith, Irvin N. Booysen, Allen Mambanda

**Affiliations:** 1 School of Agriculture and Science, University of KwaZulu-Natal, Private Bag X01, Scottsville, Pietermaritzburg, 3209 South Africa; 2 Department of Human Biology, Faculty of Health Science, Observatory, University of Cape Town, Cape Town, 7925 South Africa; 3 Department of Chemistry, University of Cape Town, Private Bag X3, Rondebosch, 7701 South Africa

**Keywords:** Ga(III) complexes, Substitution kinetics, DNA/BSA interactions, Molecular docking, Anticancer cytotoxicity

## Abstract

Octahedral iodo gallium(III) complexes, GaL1-L3(SN)I, incorporating bis-(azaaryl)amine ligands (L1–L3) with varying conformational flexibility, were synthesized and characterized by spectroscopic and elemental analyses to evaluate ligand effects on reactivity and affinity for biological targets. Iodide substitution by biologically relevant nucleophiles (guanine and thiourea), monitored spectrophotometrically, revealed ligand-dependent rates, with the fastest substitution observed for the dicationic GaL1(SN)I bearing the most compact and flexible ligand (L1), compared to the more rigid monocationic L2 and L3 analogues. UV–Visible studies of interactions with bovine serum albumin and calf thymus DNA indicated moderate to strong binding (*K*_b_ ≈ 10^4^ M⁻^1^), with affinities decreasing in the order GaL1(SN)I > GaL2(SN)I > GaL3(SN)I. Competitive binding assays, supported by molecular docking, suggested bimodal binding modes involving moderate groove binding and partial intercalation. Density functional theory calculations demonstrated that trends in structural parameters and electronic reactivity descriptors were consistent with experimentally observed iodide substitution rates and biomolecular binding affinities. Cytotoxicity evaluation of L1–L3 and GaL1-L3(SN)I against selected human cancer and non-malignant cell lines demonstrated moderate antiproliferative activity. Notably, L1 and GaL1(SN)I exhibited enhanced inhibition comparable to cisplatin against HeLa cervical cancer cells, highlighting their therapeutic potential. The data provided insights into the structural-activity relationships governing the reactivity and anticancer activity of Ga(III) complexes bearing N^∧^N^∧^N and thiosemicarbazide chelators.

**Supplementary Information:**

The online version contains supplementary material available at 10.1007/s11144-026-03129-6.

## Introduction

Octahedral gallium(III) complexes stabilized by suitable chelate ligands to reduce the toxicity of Ga(III) ions have emerged as promising alternative anticancer therapeutics. Sharing the same formal charge, coupled to a comparable ionic radius and coordination geometry, Ga(III) ions are potential metabolic mimics for Fe(III) in ion-mediated cellular processes [1, 2]. The hyper-growing cancer cells are characterized by high oxygen demand and Fe(III) absorption [3, 4], for which administered Ga(III) ions or complexes can mimic without the body rapidly excreting them, enhancing extravasation and eventual absorption into the cells [5]. This can be tapped into as a useful therapeutic effect of Ga(III) complexes, leading to the antiproliferation of the rapidly growing cancer cells.

The side effects of Pt(II) drugs such as cisplatin and its analogues [6–8], which include dose-limiting nephrotoxicity, ototoxicity, and the development of drug resistance in certain tumor cell lines [9–12], have inspired the broader search for novel metallo-chemotherapeutics with reduced toxicity to healthy cells and improved efficacy. Consequently, this search has expanded to include alternative complexes of other metal ions [12–14]. Preferably, these complexes should have distinct mechanisms of action, be active in a wide range of tumors, and be less toxic to healthy cells to allow administration of appropriate low dosages for effective therapy.

Cancer screening studies have shown Ga(III) salts (nitrates, chlorides, maltolates) to have antineoplastic activity, particularly against non‐Hodgkin’s lymphoma and urothelial carcinoma [15, 16]. However, their anticancer animal model studies were stunted by rapid hydrolysis, poor pharmacokinetics, rapid clearance, and limited bioavailability in cancer cells [17, 18]. Sadly, gallium nitrate in particular is rapidly cleared from the body, necessitating frequent high-dose infusion to maintain cytotoxic equilibrium concentrations in tumor cells and tissues [19]. Despite these pharmacodynamic shortcomings of the Ga(III) salts, there has been a development of better stabilized octahedral Ga(III) complexes that show controlled reactivity and selectivity indices, thus spurring a metabolic chemotherapeutic advantage over the clinically approved Pt(II) anticancer drugs. The closed valence shell of Ga(III) precludes rapid and indiscriminate generation of reactive oxygen species in healthy tissues, and hence reduces systematic toxicity [20]. Just like Fe(III) ions, Ga(III) complexes are preferentially adsorbed onto the receptors of transporter proteins such as transferrins and albumins in the bloodstream, and efficiently absorbed across cell membranes, thereby facilitating their target delivery in tumor cells where they can be mistaken as Fe(III) ions [21, 22]. This enhances the cytotoxic efficacy and selectivity for the tumor cells by the Ga(III) complexes while sparing normal cells. In addition, by mimicking Fe(III) in iron‐dependent metabolic processes and their regulating enzymes, such as the ribonucleotide reductases, Ga(III) complexes have the potential to kill cancer cells by a mechanism distinct from that of conventional Pt(II) complexes [23–25].

Pincer N^∧^N^∧^N ligands with π-conjugated moieties within the chelate framework have the potential to stabilize octahedral complexes of Ga(III) in both aqueous and biological environments, by forming complexes with a balanced hydrolytic stability and inertness towards nucleophilic ligand exchange [26]. *Bis*-(azaaryl)amine (N^∧^N^∧^N) ligands are interesting tridentate metal chelators. Depending on the rigidity of these ligands, they can bind the metal ion meridionally in the equatorial plane or with the donor atoms located in two orthogonal planes enabled by the presence of a flexible spacer group on one or both arms of the tridentate chelates. These spacers are typically methylene or ethylene linking groups and allow the ligands to coordinate in a folded *syn*-donor conformation mode [27–30]. These two coordination modes present variance in the steric influence within the spherical octahedral geometry. Another notable difference is that the amine N-donor atom of the rigid, *bis*-(azaaryl)amine ligands has to deprotonate first before the ligand’s chelation can take place [31, 32]. However, the presence of methylene arm stabilizes the mixed secondary (arylmethyl)aryl)amine as in the structure of N-(quinolin-8-ylmethyl)quinolin-8-amine, allowing direct chelation without prior deprotonation [27–30]. The aromatic moieties of the N^∧^N^∧^N ligands foster ligand rigidity that modulates spatial steric demands as well as π-acceptability and electrophilicity control of the metal centre. Additionally, the electron density at the metal centre can be attenuated by substituents with varying electron-donating or withdrawing abilities [33]. All these structural factors can be manipulated to optimize the lipophilicity, redox properties, protein‐binding affinities, and reactivity of the octahedral complexes towards nucleophilic ligand exchange reactions. An optimum aqueous lipophilicity balance enhances cell membrane absorption in serum transporter proteins, improves activation, pharmacodynamic and kinetic profiles, ensures selective and targeted delivery in tumor cells, as well as cytotoxicity of the candidate drugs [34].

Inclusion of a bidentate ligand in the octahedral geometry of Ga(III) ions further modulates lipophilicity and steric control around the metal centre. Convenient candidates for this role are thiosemicarbazide ligands. They coordinate metal ions bidentately as N^∧^S (S, N) donor ligands, forming octahedral Ga(III) complexes with strong Ga─S/N bonds, incorporated into five or six-membered metal-cycle chelates. These complexes are thermodynamically and kinetically stable, and have enhanced distribution and pharmacodynamic profiles that ensure better cytotoxic potential and reduced systemic toxicity [35]. So, for octahedral Ga(III) complexes combining the rigid pincer *bis*-(azaaryl)amine N^∧^N^∧^N-donor sets in the equatorial plane and an N^∧^S donor set of the thiosemicarbazide in the *facial* plane, the eighth position of the coordination sphere is conveniently occupied by a good leaving group such as a halide or aqua ligand. This ensures a mixed mode of interaction at the site of therapy. Moreover, thiosemicarbazide’s S, N-donor chelate further modulates the electronic distribution around the Ga(III) centre through a ligand-to-metal charge transfer, and this can be beneficial to the reactivity and biological activity of the complexes. Also, noteworthy is that some derivatives of thiosemicarbazones exhibit anticancer, antibacterial, and antiviral properties [36, 37]. Such anticancer activity of a coordinated ligand has the potential to amplify cytotoxic effects of resultant Ga(III) complexes against cancer cells. Ga(III) complexes bearing thiosemicarbazide or their Schiff bases (thiosemicarbazones) have been reported to exhibit improved cellular uptake, selective accumulation in malignant tissues, and inhibit some enzymatic pro-cancer cellular processes that are regulated by metal-dependent reductases such as ribonucleotide reductase [38–40].

**Fig. 1:**
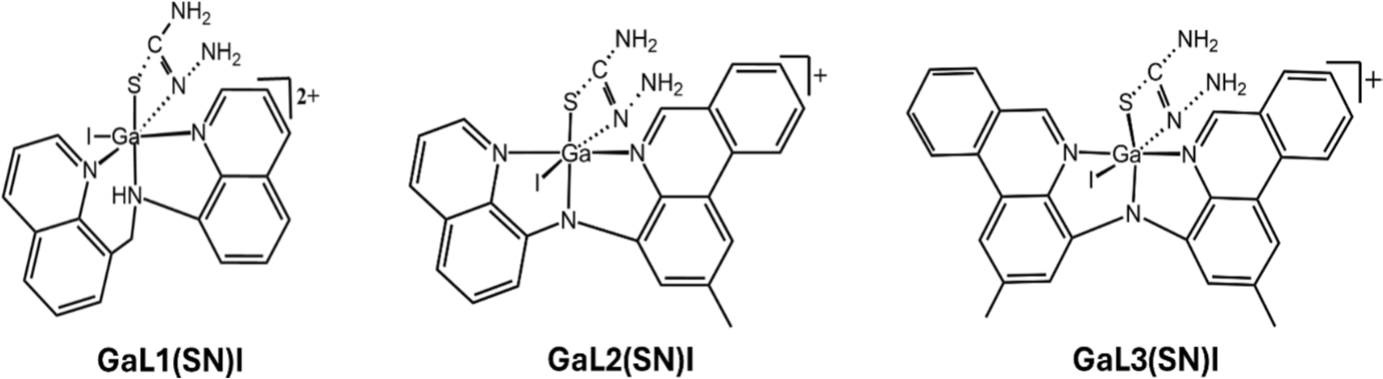
Structures of octahedral [Ga(III)(N^∧^N^∧^N)(SN)I]^2+/+^ complexes, N^∧^N^∧^N = *bis*-(azaaryl)amine and S.^∧^N = thiosemicarbazide, n = 1 or 2

Herein, we have synthesized three octahedral iodo Ga(III) complexes tethered with *bis*-(azaaryl)amine (N^∧^N^∧^N) ligands (azaaryl = quinoline, phenanthridine) and thiosemicarbazide (S, N) chelate ligands, their structures are shown in Fig. 1. The aqueous stability, reactivity towards two biogenic N- or S-nucleophiles, biological interactions with DNA/BSA, and in vitro cytotoxicity in selected (breast, pancreatic, and cervical) cancer cell lines were studied. The molecular docking of complexes onto the receptors of DNA/BSA and some cancer-associated proteins was also undertaken.

## Experimental

### Materials and methods

Reagents for the synthesis of intermediates, the bis-(azaaryl)amine ligands (L1-L3), and the corresponding octahedral complexes, as well as those used in kinetic measurements and biological evaluations, are listed in the Supplementary section.

### Instrumentation

NMR spectra of ligands and complexes (in CDCl₃) were collected on a Bruker 400 MHz spectrometer. FT-IR measurements were recorded (in the range 4000–400 cm^−1^) with a Bruker Alpha FT-IR equipped with an ATR Platinum Diamond accessory. UV–Visible absorption data were obtained on an Agilent Cary 60 spectrophotometer. High-resolution mass spectra were acquired on a Waters Micromass LCT Premier instrument using electrospray ionization and a TOF mass analyser. Fluorescence emission and excitation spectra of the samples were recorded in a 1 cm quartz cell holder on a Perkin Elmer LS-45 spectrometer with a xenon lamp. Elemental (CHNS) analyses were performed on a Thermo Scientific. Flash 2000 analyser. A Glomax® microplate spectrophotometer was used to read absorbance for the cell viability assay at 600 nm.

### Synthesis of N^∧^N^∧^N chelate ligands

The precursors for the synthesis of the *bis*-(azaaryl)amine chelate ligands (L2-L3) were all synthesized following procedures described in the literature [32]. Ligands* N*-(quinolin-8-ylmethyl)quinolin-8-amine, L1 [27], *N*-(quinolin-8—yl)-2-methylphenanthridin-4-amine (L2) [31, 32, 41] and *bis*-(2-methylphenanthridin-4—yl)amine (L3) [31, 32, 41] were synthesized following literature procedures with minor modifications. Detailed procedures and characterization data are listed in the Supplementary section.

### Synthesis of octahedral Ga(III) complexes

#### *[(Iodo)(N-(quinolin-8-ylmethyl)quinolin-8-amine)(thiosermicabazide)Ga(III)] dihexafluorophosphate (GaL1(SN)I)*.

*N*-(quinolin-8-ylmethyl)quinolin-8-amine (0.0500 g, 1.8 mmol) was dissolved in methanol (25 mL), followed by the addition of gallium(III) iodide (Ga_2_I_6_, 0.0395 g, 0.88 mmol) and sodium tert-butoxide (NaOtBu, 0.00500 g, 1.2 mmol). The mixture was refluxed for 8 h, affording an orange solution. Thiosemicarbazide (0.0159 g, 1.8 mmol) was then added slowly, and the reaction mixture was further refluxed for 4 h under a nitrogen atmosphere. To induce precipitation of the salt complex, NH_4_PF_6_ (0.00200 g, 1.9 mmol) was added towards the end of the reaction, and the stirring continued for a further 1 h. Thereafter, the solvent was removed by microfiltration under partial vacuum. The resulting orange-brown precipitate was washed sequentially with cold ethanol, diethyl ether, and dried under vacuum to give the desired product in 63% yield.

The NMR data is listed as chemical shift, (signal hyper splitting, nucleus integration number and atom numbering in the structure- for the latter refer to the SI file).

^1^H NMR (CDCl_3_, 400 MHz; δ (ppm): 9.35 (s, H, Ha); 8.61 (d, H, Hb), 8.36 (d, H, Hc), 8.09 (d, 2H, Hd), 7.90 (m, H, He), 7.75 (t, H, Hf); 7.89 (d, H, Hg); 5.15 (s, H, Hi); 3.74 (s, H, Hl); 2.64 (s, H, Hj). ^13^C NMR (100 MHz, CDCl_3_, 303 K), [δ ppm]: 153.44, 149.46, 135.92, 132.12, 131.27, 129.95, 128.93, 128.02, 127.41, 122.42, 114.05, 110.67, 105.32, 65.69, 44.38, 32.14, 30.06, 23.58, 22.29, 14.71). IR (cm^−1^): 3402 ν(NH), 1704 ν(C = C), 1486 ν(C = N), 1173 ν(C-N), 2318 ν(C-N–C), 2918 ν(C-N–C), 3702 ν(NH), 750 ν(aromatic C-H vibrations). ESI^+^-MS: calculated for [C_20_H_18_N_6_ISGa]^2+^ (569.9203). Found: 569.0682 [M]^+^. EA calculated [C_20_H_18_N_6_ISGa](PF_6_): C 33.55; N 11.74; H 2.53, Found: C 33.19; N 11.47; H 2.80.

GaL2(SN)I and GaL3(SN)I were synthesized following a similar procedure described for GaL1(SN)I.

#### *[(Iodo)(N-(quinolin-8—yl)-2-methylphenanthridin-4-amido)(thiosermicabazone)Ga(III)] hexafluorophosphate (GaL2(SN)I)*

^1^H NMR (CDCl_3_, 400 MHz; δ (ppm): 9.31 (s, H, Ha); 8.59 (d, H, Hb), 8.35 (d, H, Hc), 8.10 (t, 2H, Hd), 8.27 (m, H, He), 7.75 (t, H, Hf); 7.64 (d, H, Hg); 7.57 (t, H, Hh); 7.44 (m, H, Hk); 7.29 (t, H, Hl); 6.93 (d, H, Hn); 3.71 (d, H, Hm); 2.62 (s, H, Hj). ^13^C NMR (100 MHz, CDCl_3_, 303 K), [δ ppm]: 153.39, 147.41, 139.94, 137.76, 135.97, 134.05, 132.10, 131.26, 129.48, 128.92, 128.01, 127.36, 126.52, 125.32, 122.04, 121.67, 116.04, 110.05, 31.92, 29.69, 23.08, 21.59, 14.11, 11.72. IR (cm^−1^): 1610 ν(C = C), 1256 ν(C-CH_3_ stretch), 1447 ν(C = N), 1104 ν(C-N), 2918 ν(C-N–C), 751 ν(aromatic C-H vibrations). ESI^+^-MS: calculated for [C_24_H_20_N_6_ISGa]^+^ (619.8618). Found: 569.0682 [M]^+^. EA calculated [C_24_H_20_N_6_ISGa](PF_6_): C 37.63; N 10.97; H 2.63, Found: C 37.90; N 11.28; H 2.35.

#### [*(Iodo)(bis-(2-methylphenanthridin-4—yl)amido)(thiosermicabazone)Ga(III)] hexafluorophosphate (GaL3(SN)I)*

^1^H NMR (CDCl_3_, 400 MHz; δ (ppm): 9.08 (d, H, Ha); 8.56 (t, H, Hb), 8.13 (d, H, Hc), 8.01 (t, 2H, Hd), 7.70 (m, H, He), 7.44 (t, H, Hf); 7.25 (m, H, Hg); 6.85 (s, H, Hh); 4.45 (s, H, Hl); 2.57 (s, H, Hk); 2,28 (s, H, Hj). ^13^C NMR (100 MHz, CDCl_3_, 303 K), [δ ppm]: 153.32, 149.45, 144.75, 142.61, 138.79, 137.29, 134.06, 132.93, 132.39, 130.83, 129.49, 128.55, 127.08, 124.58, 122.37, 113.53, 111.14, 107.13, 83.08, 67.81, 38.95, 31.94, 29.71, 27.06, 23.02, 22.29, 19.51, 14.12, 11.10. IR (cm^−1^): 3382 ν(NH), 1663 ν(C = C), 1451 ν(C = N), 1173 ν(C-N), 2818 ν(C-N–C), 3702 ν(NH), 738 ν(aromatic C-H vibrations). ESI^+^-MS: calculated for [C_29_H_24_N_6_ISGa]^+^ (684.2071). Found: 685.1074 [M]^+^. EA calculated [C_29_H_24_N_6_ISGa](PF_6_): C 41.96; N 10.12; H 2.91, Found: C 42.27; N 9.85; H 3.07.

### Reactivity, stability and biological interactions of Ga(III) metal complexes

#### Stability of complexes in DMSO and buffer solution

The aqueous stability of each metal complex was assessed under simulated physiological conditions (37 °C). Solutions (0.1 mM) of each metal complex were prepared in phosphate-buffered saline (PBS) containing 2% DMSO (*v/v*), and their UV–Visible spectra were recorded hourly for 24 h. To evaluate resistance to DMSO solvolysis, the UV–Visible absorption spectra of the metal complexes dissolved in anhydrous DMSO were recorded at 1 h intervals for 24 h.

#### Computational studies

Geometry-optimized structures of Ga(III) complexes were computed at the Density Functional Theory (DFT) level using the B3LYP/LANL2DZ method in the Gaussian 09 suite programs [42]. The 6—311 core-valence basis set was applied to all non-metal atoms, while the LANL2DZ basis set, which uses effective core potentials, was applied to the metal centres [43]. In addition, time-dependent Density Functional Theory (TD-DFT) calculations were performed in DMSO, for which solvent effects on the geometry of the complexes are taken into account according to a Conductor Polarizable Continuum Model (C-PCM) [44]. The calculations were carried out to obtain geometry-optimized molecular structures, trending data on the energetics of the frontier molecular orbitals, and structural metrics such as bond angles and lengths, atomic charges, and predictive electronic reactivity descriptors.

The optimized structures of the octahedral Ga(III) complexes were also used as input structures in simulated docking experiments to gauge the strength of their non-covalent interactions and affinities for receptors of selected macromolecules such as CT-DNA and BSA. Docking onto relevant receptor sites of BSA probed the host transporter protein’s affinity and hence efficiency to transport and deliver them into cells. [45]. Molecular docking simulations were carried out using Autodock tools 1.5.7 [46]. The Autodock parameter execution files were modified by adding parameters of Ga(III) ions since the default files do not have them. The metal complexes were docked on the receptor biomolecules relevant to the treatment of cancers, viz*.*, B-DNA (PDB:1bna), bovine serum albumin (PDB:4f5s). Additionally, GaL1(SN)I and L1 (as leading antiproliferation agents) were docked onto receptors of human aromatase cytochrome P450 (PDB:3EQM) [47, 48], and peptidyl-prolyl isomerase (PDB:4DRH) [49, 50]. Details on the pre-modification of the crystal structures of the receptor macro molecules, applied structural parameters of GaL1-L3(SN)I and their free ligands were done as described in the Supplementary Information section. Docked conformers of the Ga(III) complex-receptor molecule were analysed and visualized using Bovia Discovery Studio 2024.

#### Rates and mechanism of iodide substitution from the Ga(III) complexes.

Stock solutions of GaL1-L3(SN)I complexes, and the nucleophiles thiourea (Tu) and guanine (Guan) were prepared by dissolving accurately weighed amounts of each in methanol containing 0.10 M LiI. The LiI suppresses the spontaneous solvolysis of the iodo metal complexes [51]. Solutions for kinetic analysis of the substitution reactions were prepared by diluting the stock solutions of the metal complexes to 0.1 mM, and the concentration of nucleophiles was prepared at 10–50‐fold excess to ensure pseudo-first‐order kinetic conditions. Equal volumes of metal complexes and nucleophiles (in duplicates) were thermally equilibrated (298–313 K) in tandem cuvettes in the sample holder of a UV–Visible absorption spectrophotometer. The reaction was initiated by quickly mixing the reactants and recording the absorbance of the solution as a function of time at a set wavelength. Linear plots of the concentration‐dependent observed rate constants at 298 K were used to determine the bimolecular rate constant, *k*_2,_ for the iodo substitution from each complex. Temperature-dependent rate constants of the 30-fold nucleophile solution were measured in the temperature range of 298–313 K, at 5 K increment intervals. Data of* k*_2_ were plotted to obtain Eyring plots from which the activation parameters (Δ*H*^≠^ and Δ*S*^≠^) were estimated.

#### DNA/BSA Spectroscopic titrations with the metal complexes.

Detailed DNA/BSA spectroscopic (absorption/emission) titrations to probe the binding affinities of the metal complexes are provided in the Supplementary Information section.

#### Cell culture growth, maintenance and treatments

Details on the cell culture growth, maintenance and treatments are as described in the Supplementary Information section.

#### The 3-(4,5-dimethylthiazol-2—yl)-2,5-diphenyltrazolium bromide (MTT) Cell viability assay.

To evaluate the cytotoxicity of GaL1-L3(SN)I complexes or the free ligands, L-L3, selected cell lines were treated at 10 µM (0.1% DMSO), and the viability of the cultured cells was measured using the MTT assay. Briefly, cells were plated in a 96-well plate at densities of 3 × 10^3^–1 × 10^4^ cells per well and allowed to adhere for 24–48 h. After incubation, cells were treated for 48 h with 10 μM of each ligand (L1-L3) or Ga(III) complexes, along with 10 μM CDDP and 0.1% DMSO. Thereafter, 10 μL of 5 mg/mL MTT solution was added to each well, and the plates were incubated at 37 °C for 4 h to allow the formation of the purple formazan crystals. After incubation, 100 μL of the solubilising reagent was added to each well, followed by overnight incubation to dissolve the crystals. Absorbance was measured at 600 nm using a Glomax® microplate spectrophotometer, and mean cell viability was calculated and expressed as a percentage of the vehicle control.

## Results and discussion

### Synthesis and characterization of octahedral Ga(III) complexes.

The octahedral Ga(L2-L3)(SN)I complexes were formed from a two-step, one-port synthesis. First, the intermediate complexes, Ga(L1-L3)I_3_ were prepared from equimolar reactions of the dimeric Ga_2_I_6_ precursor and two equivalents of the corresponding tridentate ligands, L1-L3, at 50 °C for 8 h in methanol under basic conditions provided by the bulk and non-coordinating NaOtBu salt. Afterwards, an equivalent of thiosemicarbazide was added to the intermediates, and the mixture was stirred for a further 4 h to afford orange-brown powders of GaL1-L3(SN)I in yields of 62–76%. Specifically, the relatively flexible ligand, L1, coordinated *facially* to the Ga(III) ion as a neutral N^∧^N^∧^N ligand with the N-donor atom of the quinolin-8-ylmethyl fragment located in a plane orthogonal to that of the quinolin-8-amine. This is possible due to the flexibility of the methylene spacer group, which allowed the 8-quinolinyl arm of the ligand to bond tridentately in a folded, *syn* manner. This coordination mode has been reported for single crystal structures of both the closed shell (Cd(L1)_2_) and the dichlorido-bridged octahedral (Cd(II)L1)_2_Cl_3_) complexes of L1, as well as in rerated structures of mono and dinuclear Ga(III) complexes with dipicolate, tris(picolate) and 1,3 diacetylated propapanediamine stabilizing ligands [27–30]. The subsequent *facial* coordination of the thiosemicarbazide resulted in a dicationic octahedral GaL1(SN)I complex with an iodo leaving group. In contrast, the rigid proligands, L2-L3’s amine N donor atoms are deprotonated by the *tert*-butoxide base to form an activated monocationic, *bis*-(azaaryl)amido analogues with N^∧^N^−∧^N donor sets. The *bis*-(azaaryl)amido ligands (L2-L3) coordinate the Ga(III) ion meridionally. *Facial* capping by the S, N-thiosemicarbazide forms the Ga(L2-L3)(SN)I complexes, bearing an iodide as the good leaving group and located in a plane orthogonal to the tridentate.

All complexes are soluble in chlorinated and high-boiling aprotic solvents such as CHCl_3_*,* DCM*,* DMSO and DMF; however, their solubility is poor in such solvents as hexane, acetone, acetonitrile, ethyl acetate, methanol and ethanol. The complexes were characterized by various spectroscopic techniques (NMR, FTIR, UV–Visible, ESI^+^-TOF-mass spectrometry and elemental analysis).

^1^H-NMR spectra of GaL1-L3(SN)I complexes show proton signals in the aromatic region that are shifted slightly upfield by 0.1—0.3 ppm relative to the free ligands, which appear as multiplets in the range 7.1–8.2 ppm (Fig. S1–S3). The ^1^H-NMR spectrum of the GaL1(SN)I confirm the presence of the amine (N–H) proton peak (which appears near 5.6 ppm in the spectra of free ligands), indicating that L1 coordinate the Ga(III) without deprotonation [27–30]. In contrast, the proton resonance is characteristically absent in the spectra of the GaL2-L3(SN)I complexes, thus confirming the deprotonation and coordination of the ligands to the Ga(III) [31, 32].In addition, the total number of carbon resonances in the ^13^C and ^1^H NMR spectra of all complexes correlates with their theoretical count. Similar spectral changes are also noted in the FTIR spectra of the complexes and the free ligands. For example, the FTIR spectra of uncoordinated ligands feature an N–H stretching band around 3380 ppm, and this band is absent in the spectra of the corresponding GaL2-L3(SN)I complexes, confirming successful deprotonation of N–H and coordination of the ligands, L2-L3, before their coordination to Ga(III) precursor (Figs. S7–S9). The ESI^+^-(TOF)-MS spectra feature isotopic patterns characteristic of the theoretical masses and elemental compositions of the synthesized GaL1-L3(SN) complexes (Figs. S10—S12). The GaL1-L3(SN) complexes were synthesized in reasonable purity as verified by a close agreement (all below 5% margin of error) between the experimental elemental composition and the theoretically calculated values (Figs. S13–S15).

The UV–Visible absorption spectra of GaL1-L3(SN)I complexes show intense absorption bands in the 260—320 nm region, which are assigned to ligand-centred (LC) π → π* transitions within the aromatic fragments of L1-L3 and multi-bonds within the thiosemicarbazide framework. The additional weak shoulders extending from 320 to 370 nm are consistent with intraligand charge-transfer (ILCT) from the tridentate ligands’ (L1-L3) amino bridges into the π* MOs of the flanking near-planar aromatic moieties (Figs. S16 and S17). In general, metal-to-ligand charge-transfer (MLCT) transitions are absent or weak for octahedral Ga(III) complexes. This is because Ga(III) is a closed-shell d^10^ ion, and the only vacant atomic orbitals are the relatively high-energy lying *f*-orbitals. Therefore, it has no low-energy and partially occupied metal *d*-orbital from which electron density can be back-donated into appropriate ligand-centred π*-orbitals. However, in the spectra of complexes of lL1-L3**l,** a common low absorption band is observed above 400 nm. This may be due to a weak ligand-to-metal charge-transfer (LMCT) transition involving electron donation from the amido of the N^∧^N^∧^N ligand or thioamide sulphur of the thiosemicarbazide into low-energy lying unoccupied Ga(III) orbitals. The experimental bands sharply contrast those predicted by TD-DFT computations. The simulated UV–visible absorption spectra exhibit higher molar extinction bands in the visible range, 480–520 nm, that extend as low absorption edges into the near-infrared range. High-energy associated IL bands are accurately predicted in the computed spectra; however, the intensity of these bands is significantly underestimated compared to the experimentally observed transitions. Conventional hybrid gradient functionals, such as B3LYP, tend to over-stabilize charge-transfer states, causing an underestimation of their excitation energies, leading to artificially red-shifted CT-bands, and appearance of low-energy absorption bands in the simulated spectra [52, 53]. Moreover, for TD-DFT calculated spectra simulated in the gaseous phase, solvent-specific interactions, vibronic coupling, and tautomeric effects are assumed to be absent, and this oversimplification likely contributes to the mismatch in band intensities and distribution. Similar comparative discrepancies are also reported in the literature for octahedral Ga(III) complexes [54]. Despite these differences, both the experimental and theoretical data are comparable with respect to the occurrence of the higher-energy IL charge transfer.

### Stability of GaL1-L3(SN)I in DMSO and aqueous solution.

Time‐dependent UV–Visible spectrophotometric measurements to assess the stability of GaL1-L3(SN)I under conditions relevant to biological assays were carried out. Solutions of each complex were prepared in a 98:2 (v/v) phosphate‐buffered saline (PBS): DMSO mixture, as well as in pure DMSO, to mimic the solvent environments used in *in-vitro* cytotoxicity and biomolecular studies. Electronic absorption spectra were acquired at 37 °C for a 24 h interval, with spectral scans collected every hour to monitor any changes in the characteristic absorption bands. The UV–Visible absorption spectra of each complex, recorded in DMSO, remained essentially unchanged throughout the monitoring period, indicating that the iodo complexes exhibit high solution stability and preserve their structural integrity under these conditions [55, 56] (Figs. S18–S20).

However, in the PBS: DMSO (98:2, v/v) solvent system, there are time-dependent spectral changes characterized by a progressive attenuation of the ligand-centred absorption bands. In addition, the spectrum of GaL1(SN)I has an isosbestic point at 420 nm, indicating the formation of a new species which is probably an aqua derivative. Over time, the coordinated iodide ligands are gradually hydrolysed and substituted by an aqua ligand from the aqueous buffer, forming more reactive aqua species [57] (Figs.  S18–S20). Thus, the aqueous stability of the GaL1-L3(SN)I complexes should significantly influence their biological activity and overall in vivo reactivity.

### DFT-optimized structures and data of the GaL1-L3(SN)I complexes.

**Scheme. 1:**
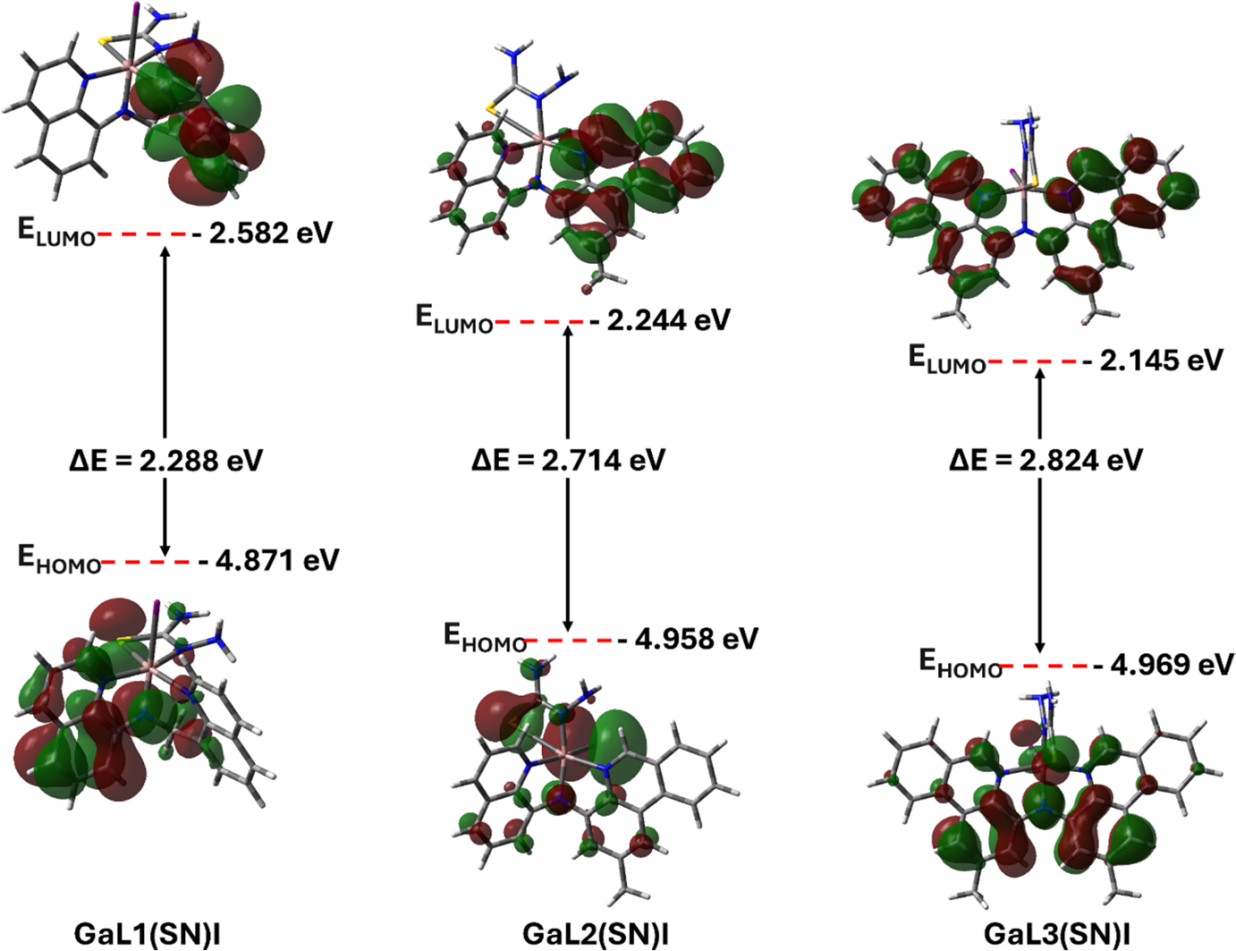
HOMO/LUMO iso-electron density surfaces, of GaL1-L3(SN)I, energy levels and the band gap energies ΔE

Density functional theory (DFT) calculations were performed to elucidate the electron distribution and structure–reactivity metrics of the octahedral Ga(III) complexes. The DFT-optimized structures and calculated HOMO–LUMO frontier orbitals of the Ga(III) complexes are presented in Scheme 1.

All the optimized structures of complexes have a distorted rigid octahedral geometry. Analysis of both the HOMO (Highest Occupied Molecular Orbital) and LUMO (Highest Unoccupied Molecular Orbital) distributions shows that they are predominantly localized on the π-motifs of the chelate ligands, with minimal to no contribution from the Ga(III) ion. The HOMO orbital density is primarily localized on the donor ring of the flanking aromatic rings of the *bis*-(azaaryl)amine/amido N^∧^N^∧^N chelators, as well as on the sulfur atom and the adjacent nitrogen atoms of thiosemicarbazide. In contrast, the LUMO is predominantly mapped over the inner π-orbitals of the N^∧^N^∧^N ligands, with negligible contributions from the lone pairs of the thione or imine group on the thiosemicarbazide moiety*.* This suggests that the complex’s redox activity and nucleophilic behavior are likely governed by motifs on the ligand framework rather than the metal centre. Moreover, the pronounced localization of the HOMO on the ligand motifs suggests that nucleophilic reactivity is governed by electron donation from the sulphur and nitrogen towards electrophilic centres. Given the minimal contribution of Ga(III) centred orbitals to the HOMO or LUMO, these electron transitions are dominated by intraligand π-π* within each chelated ligand or weak ligand‐to‐ligand charge transfer processes. The molecular electrostatic potential surfaces (MEPS) mappings (Fig. S21) have a uniform green colored charge distribution, meaning that all the complexes are kinetically inert towards nucleophilic substitution.

The effects of structurally modifying quinoline through benzannulation and introduction of methylene groups are evident in the DFT calculated bond angles, lengths, atomic charges, band gap energies, and reactivity parameters. GaL1(SN)I exhibit longer Ga─I bond and a wider bite angle of the tridentate ligand (2.949 Å, 160.04°) due to its six-membered chelate formed by the flexible quinolin-8-ylmethyl arm of L1, compared to the amido analogues that incorporate the rigid phenanthridine chelates for GaL2(SN)I (2.822 Å, 112.25°) and GaL3(SN)I (2.745 Å, 104.36°) both in the equatorial plane. The calculated relative energies are illustrated in Scheme 1, and values of the energy band gaps are presented in Table 1. They increase in the order: GaL1(SN)I (2.288 eV) < GaL2(SN)I (2.714 eV) ≈ GaL3(SN)I (2.824 eV), and correlate with the increasing rigidity, extension of π-conjugation, and the introduction of methyl substituents. The trend of computed electrophilicity indices (ω) of the complexes increases in the order GaL3(SN)I < GaL2(SN)I < GaL1(SN)I. These trends predict that GaL1(SN)I complex has a lower activation energy, therefore will exhibit higher chemical reactivity in comparison to the L2 and L3 analogues.

**Table 1:** Summary of DFT-calculated data for the optimized structures of octahedral GaL1-L3(SN)I complexes

		Complex	
	GaL1(SN)I	GaL2(SN)I	GaL3(SN)I
Property			
Bond lengths (Å)			
Ga─I	2.949	2.822	2.745
Ga─N_1_	1.915	1.953	1.967
Ga─N_2_	2.084	2.167	2.058
Ga─N_3_	2.257	2.061	2.065
Bond angles (°)			
N_ 1_─Ga─N_2_	51.93	54.62	50.83
N_ 2_─Ga─N_3_	160.04	112.25	104.36
N_ 1_─Ga─N_3_	54.26	47.28	51.10
Natural charges			
Ga	1.056	1.069	0.962
I	− 0.497	− 0.452	− 0.414
N_1_	− 0.503	− 0.582	− 0.550
N_2_	− 0.315	− 0.277	− 0.295
N_3_	− 0.361	− 0.332	− 0.290
N4	− 0.248	− 0.346	− 0.384
Dipole moment (Debye)	9.4617	9.7169	9.8415
HOMO (eV)	− 4.871	− 4.958	− 4.969
LUMO (eV)	− 2.582	− 2.244	− 2.145
ΔE (eV)	2.288	2.714	2.824
μ	− 3.727	− 3.563	− 3.569
η	1.144	1.395	1.401
ω	6.069	4.549	4.544

### Iodide substitution from GaL1-L3(SN)I by model bio nucleophiles (guanine & thiourea).

To gauge the lability of the coordinated iodide, its rate of substitution from octahedral GaL1-L3(SN)I complexes by guanine (Guan) and thiourea (Tu) nucleophiles was measured under *pseudo-*first order conditions. The octahedral Ga(III) complexes bear variable N^∧^N^∧^N *bis*-(azaaryl)amine proligands incorporating quinoline or phenanthridine fragments in the equatorial plane and facially coordinated thiosemicarbazide moieties as auxiliary S, N-bidentate non-leaving ligands. The eighth position is occupied by a labile iodide as a co-ligand. The phenanthridine arms offer an extended π-surface and conjugation in the spectator framework when compared to quinoline, which has only two aromatic rings. In addition, there are methyl substituent groups at the meta positions of the phenanthridine rings, which are bonded to the amido N. We believe that these structural modifications within the octahedral coordination sphere enact variable steric and electronic effects that affect the rate at which the iodo ligand is substituted from the octahedral coordination sphere by nucleophiles.

Experiments to monitor the rate of ligand exchange were initiated by manually mixing equi-volume of pre-equilibrated solutions of the complexes and the nucleophiles (Guan/Tu) in the concentration [Nu] range that was 10–50-fold higher than the complexes in a tandem cuvette before recording the absorption spectra (within the range 200–800 nm) as a function of time. The predetermined wavelengths for monitoring the rate of iodo substitution from the complexes are presented in Table S1. The observed first order rate constants (*k*_obs_​) for reactions of the complexes with each nucleophile were determined by fitting the kinetic absorbance data to first order exponential decay functions, as shown in Figs. S22–S23.

**Fig. 2:**
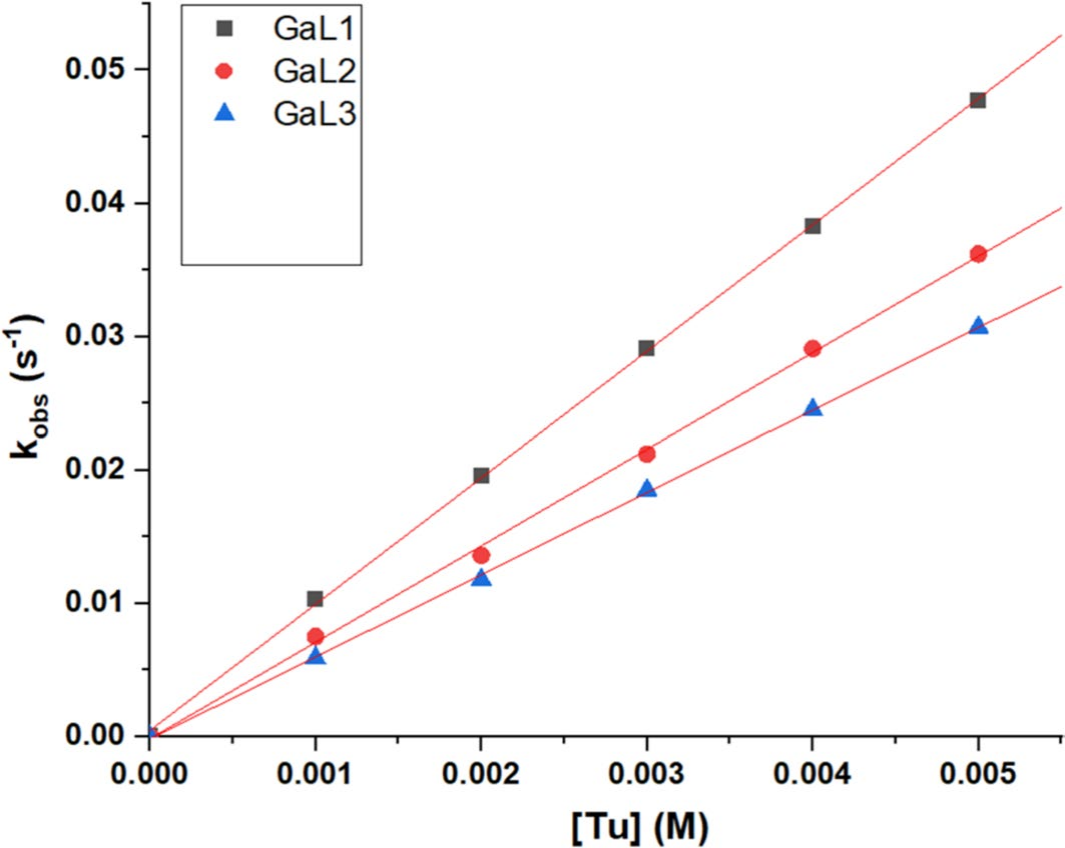
Concentration dependence plot of (*k*_obs_, s^−1^) for the iodide substitution from GaL1-L3(SN)I by Tu, *I* = 0.1 M (LiI) in methanol, *T* = 298 K

The* k*_obs_ values were determined as a function of [Nu] and temperature. The *k*_obs_ measured at variable nucleophile concentration, [Nu], were plotted linearly as depicted in Fig. 2 and S24. All linear *k*_obs_—[Nu] plots have near-zero intercept, an indication that the substitution of the iodide ligand occurred irreversibly and without the participation of the solvent species. The bimolecular rate constants, *k*_2_, (M^−1^ s^−1^), were deduced from the slopes of the plots, and their values are presented in Table 2.

**Table 2:** Second order rate constants, *k*_2_ (M^−1^ s^−1^), for the iodide substitution from GaL1-L3(SN)I by Guan and Tu at 298 K

Complex	Tu	Guan
	*k*_2_ (M^−1^ s^−1^)	*k*_2_ (M^−1^ s^−1^)
GaL1(SN)I	8.24 ± 0.2	0.83 ± 0.03
GaL2(SN)I	5.72 ± 0.2	0.70 ± 0.09
GaL3(SN)I	4.19 ± 0.1	0.58 ± 0.07

The rate constants in Table 2 for iodide substitution from octahedral GaL1-L3(SN)I complexes fall within narrow ranges for both nucleophiles and decrease in the order: GaL3(SN)I < GaL2(SN)I < GaL1(SN)I. The observed trend of the rates of substitution is attributable to both electronic and steric changes of the two chelating ligands. The contribution to the rate of ligand exchange of the S, N chelate is constant in all complexes. The relatively smaller and less sterically encumbered ligand structure of L1 minimizes congestion in the equatorial coordination plane, thereby allowing nucleophiles to approach the metal centre of GaL1(SN)I more readily. In contrast, the more rigid L2 and L3, which bear extended π-conjugated rings, increase the steric hindrance within the equatorial plane, thereby restricting mutual conformational adaptability between the two chelate ligands. This increased steric demand within the spherical octahedral geometry retards the approach of the incoming nucleophiles, and hence lowers the rate of substitution when compared to GaL1(SN)I. For the same reason, GaL3(SN)I has the lowest rate constants for iodide substitution. In addition, the rates are also affected by the variable methyl substituents’ σ-induction donation into the phenanthridine arms of the ligand in GaL2(SN)I and GaL3(SN)I. This electron donation dampens the π*-acceptor ability of L2/L3. The electrophilicity of their Ga(III) ions is lowered, leading to weaker nucleophilic attack by the incoming nucleophiles (Tu/Guan). Furthermore, the formal charge of the dicationic [GaL1(SN)I]^2+^ (a complex which bears a neutral bis(azaaryl)amine ligand) is one unit higher than the charge on the monocationic [GaL2/L3(SN)I]^+^ analogues bearing the bis(azaaryl)amido ligands. The higher positive charge of [GaL1(SN)I]^2+^ enhances its ion–dipole interactions with nucleophiles such as thiourea or guanine, effectively lowering the activation barrier for iodide substitution [58]. Thus, [GaL1(SN)I]^2+^ is more prone to nucleophilic attack than [GaL2/L3(SN)I] and hence the higher observed rates of ligand exchange in the former.

The observed reactivity trend can be further rationalized by DFT-simulated structural features of GaL1(SN)I complex, specifically the elongated Ga–I bond and the wider N–M–N bite angles. These geometrical distortions reduce the extent of metal–ligand orbital overlap, thereby lowering the activation barrier for iodide dissociation and ultimately enhancing the rate of substitution reactions. This structural rationale is consistent with the higher electrophilicity indices and lower HOMO–LUMO energy gaps obtained from DFT calculations for the L1 complex relative to its L2 and L3 analogues (Table 1). The narrower energy gap and larger electrophilicity index imply greater chemical reactivity and reduced kinetic stability for the L1 complex [59–61]. The comparatively faster substitution rates of L1 and L2 analogues are also supported by higher calculated NPA charges on their metal centres, suggesting that they are more susceptible to nucleophilic attack compared to the L1 complex with a less positively charged metal centre.

In addition, the nature of the incoming nucleophile strongly influenced the substitution kinetics. Notably, the sulphur-donor nucleophile (Tu) displaced the labile iodide ligand significantly faster than the nitrogen-donor (Guan), with reactivity factors exceeding 10. This enhanced reactivity can be attributed to the greater polarizability and larger atomic radius of sulfur donor atom, which confer a stronger affinity for coordination to the Ga(III) centre compared to the less polarizable nitrogen donor [62]. The relatively low iodide substitution rates observed for Ga(III) complexes can be linked to the strong electron-donating nature of the sulphur and nitrogen donor atoms within the thiosemicarbazone moiety. These donor groups enhance the electron density at the metal centre, thereby decreasing its electrophilicity and consequently reducing its susceptibility to nucleophilic attack. This electronic stabilization renders the complexes kinetically inert.

The temperature dependence of the second order rate constant (*k*₂) was evaluated by monitoring the rate of iodide substitution within the temperature range of 298–313 K, while maintaining a constant 30-fold excess of nucleophile relative to the Ga(III) complex. The correlation between the rate constants and temperature was analysed using the Eyring equation, from which the activation parameters were derived. Specifically, the activation enthalpy (Δ*H*^≠^) and activation entropy (Δ*S*^≠^) were obtained from the slopes and intercepts of the linearized Eyring plots. The activation enthalpy offers insights into the energetic barrier that must be overcome during the substitution reaction, reflecting the bond reorganization and electronic rearrangements required for the transition state to form. Associated with that analysis is the activation entropy, which is a measure of the change in degree of disorder that occurs to transform reactants into a transition state complex. Its magnitude sheds light on the compactness or degree of bonding of the transition state, and hence the mechanistic aspects of the reaction pathway [63]. Together, these parameters allow an understanding of the thermal energy and entropic contributions to the reactivity of the Ga(III) complexes. The calculated thermodynamic parameters Δ*H*^≠^ and Δ*S*^≠^ are presented in Table 3, and the corresponding Eyring plots are depicted in Fig. S25. The very negative activation entropies (ΔS^≠^  < 0, relatively low activation enthalpies (Δ*H*^≠^), and very negative free Gibbs’ energy of activation for the iodide substitutions support nucleophilic attack of the octahedral Ga(III) complexes by the incoming nucleophiles, and in all cases, it occurs spontaneously.

**Table 3:** Activation parameters for chloride substitution from GaL1-L3(SN)I by nucleophiles (Tu and Guan)

Nucleophile						
Tu (S-donor)				Guan (N donor)		
Complex	ΔH^≠^ kJ mol^−1^	ΔS^≠^ J K^−1^ mol^−1^	ΔG^≠^ kJ mol^−1^	ΔH^≠^ kJ mol^−1^	ΔS^≠^ J K^−1^ mol^−1^	ΔG^≠^ kJ mol^−1^
GaL1(SN)I	26 ± 1	− 189 ± 2	− 71 ± 3	40 ± 1	− 146 ± 2	− 32 ± 3
GaL2(SN)I	23 ± 1	− 171 ± 5	− 62 ± 2	44 ± 1	− 87 ± 5	− 49 ± 3
GaL3(SN)I	28 ± 2	− 136 ± 2	− 54 ± 3	47 ± 2	− 116 ± 2	− 43 ± 1

### Molecular docking of GaL1-L3(SN)I on selected receptive biomolecules

Computational molecular docking of GaL1-L3(SN)I on DNA/BSA receptors was conducted to get insight into their interactions or affinities with the host macromolecules. Simulating the molecular binding by docking them on the receptors of biological targets provides insightful information on the interactions of drugs (the ligands) and the receptors (hosts) of biomolecular targets. Physicochemical data, such as the binding affinity, interaction energies, and the specific amino acid residues involved in drug recognition and stabilization, are obtained [64]. Data from molecular docking simulations on DNA/BSA is valuable in the screening, predictive design and development of therapeutic drugs.

**Fig. 3:**
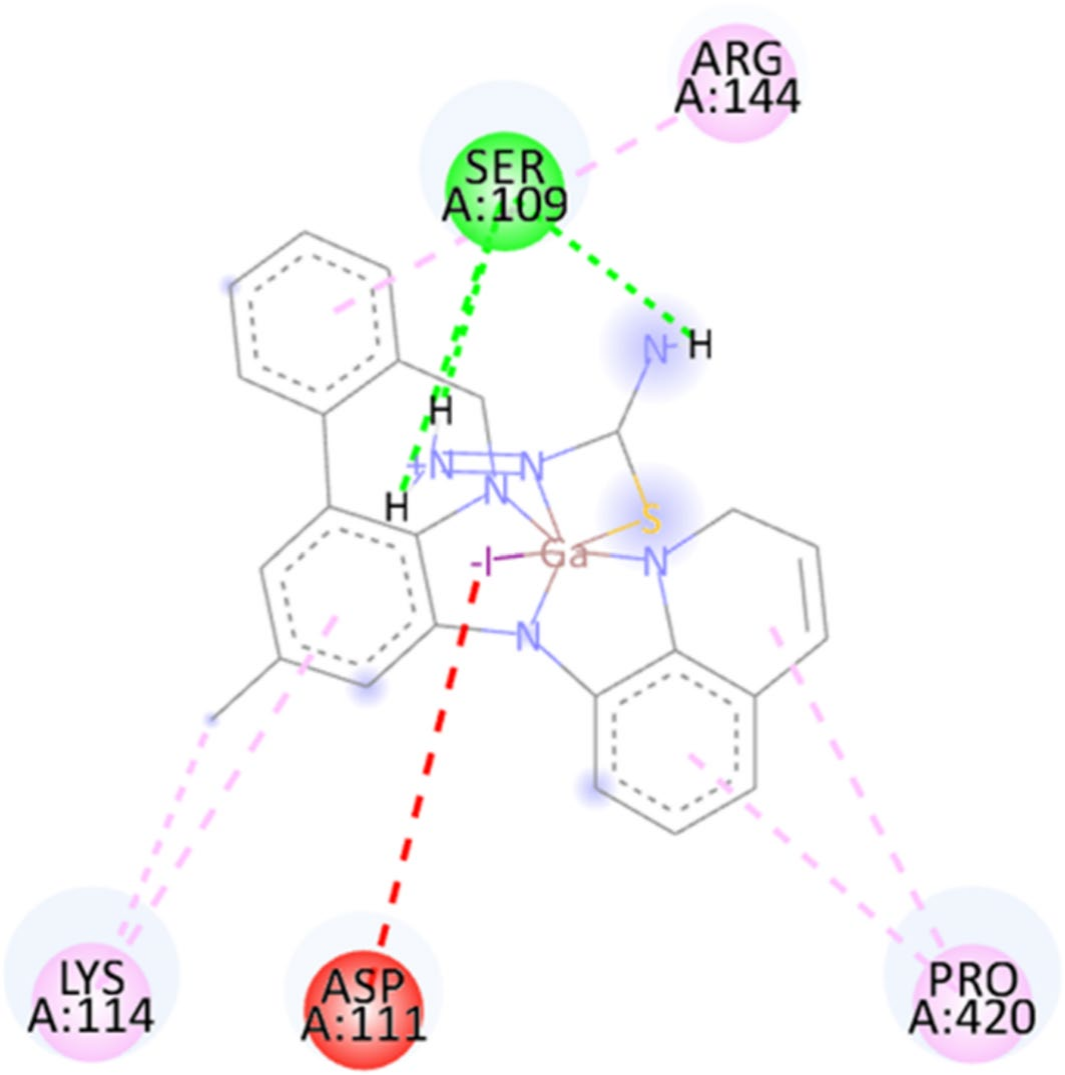
2D simulated image of GaL2(SN)I localized non-covalent bonding to BSA’s amino acid residues

Figs. 3, S26 and S27 provide examples of the simulated docking poses of GaL1-L3(SN)I and BSA. All the metal complexes exhibited a predominant binding preference for site II, located within subdomain IIIA of BSA. The non-covalent interactions are primarily established between the amino acid residues (Ala, Pro, Gly, Lys, His, Arg) of the proteins and the structural features of the metal complexes, such as the aromatic moieties, nitrogen donor atoms, and the labile iodide ligand. The calculated binding energy scores of the complexes follow the order: GaL3(SN)I (− 5.74 eV) < GaL2(SN)I (− 6.59 eV) < GaL1(SN)I (− 7.63 eV). These are higher than those reported for an octahedral Ga(III) complex coordinated with 8-hydroxyquinoline, a complex that has exhibited promising cytotoxicity [54]. A stronger binding affinity for BSA site II means higher stability of the bound form of the drug onto the albumins and prolonged biological circulation stability, both leading to more efficient drug transportation and delivery to the sites of therapy in target cells [65]. Consequently, therapy threshold equilibrium concentrations are maintained in the cancerous tissues, while concurrently minimising off-target distribution and associated toxic effects.

**Fig. 4:**
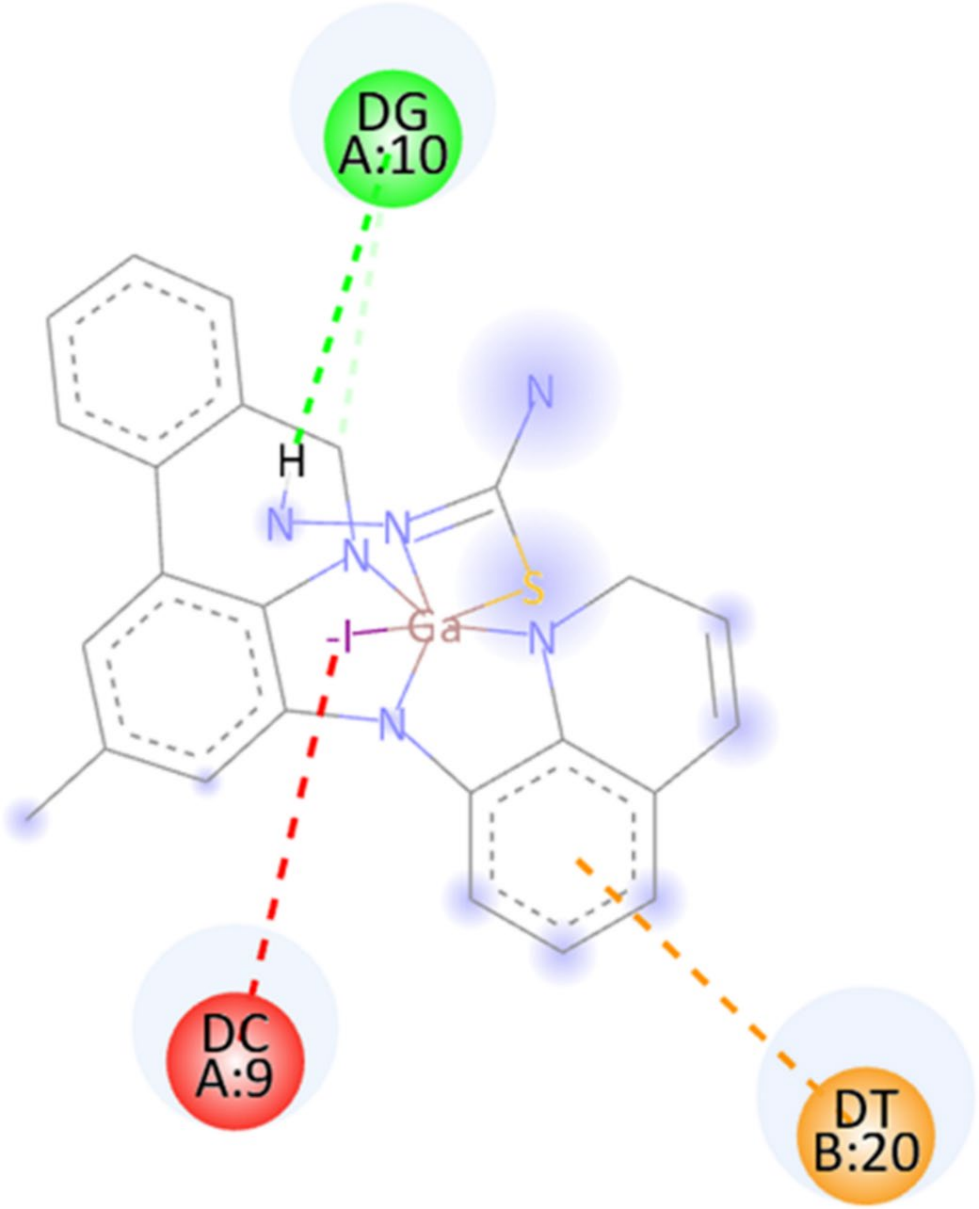
2D simulated image showing key intermolecular short contact bonds in the non-covalent interactions of GaL2(SN)I with the N-bases of DNA

GaL1-L3(SN) complexes were also docked onto DNA to assess their binding affinities and elucidate the specific non-covalent interactions involved in the binding process. The simulated binding poses depicted in Figs. 4 and S28 and S29 show that the aromatic moieties, nitrogen atoms, and iodide ligand of the complexes engage in non-covalent interactions with nucleobases (adenine, thymine, guanine, and cytosine) in the minor grooves of DNA. The computed binding affinities scores and the stability of the formed DNA-metal complex adducts are summarized in Table 4, and they decrease in the order: GaL1(SN)I (9.57 eV) > GaL2(SN)I (7.59 eV) > GaL3(SN)I (7.00 eV).

**Table 4:** Calculated stability scores (eV.) for non-covalent binding of GaL1-L3(SN)I complexes at the preferred sites of DNA and BSA receptors

Complex	BSA binding scores (eV)	RMSD (Å) of the pose	DNA binding scores (eV)	RMSD (Å) of the pose
GaL1(SN)I	− 7.63	0.491	− 9.57	1.75
GaL2(SN)I	− 6.59	0.604	− 7.59	1.54
GaL3(SN)I	− 5.74	0.353	− 7.00	0.308

The lead compounds GaL1(SN)I and L1, which demonstrated promising inhibitory activity in the cytotoxicity assay (vide infra), were further docked into receptors of peptidyl-prolyl isomerase (4DRH) and cytochrome P450 19A1 (3EQM) proteins, which promote the progression and proliferation of breast and cervical cancers [66, 67]. Their simulated binding interactions and poses are depicted in Fig. S30 and S31, while the computed binding affinity constants are summarized in Table 5. GaL1(SN)I and its ligand, L1, exhibited moderate to strong binding affinities for the receptors of the target proteins. These favorable interactions suggest that the two compounds may exert anticancer activity through LI-assisted binding and inhibiting the activity of the associated cancer-regulating proteins, thereby providing alternative mechanisms to DNA covalent bonding displayed by cisplatin and analogues. This could potentially overcome drug resistance in certain cell lines and minimize off-target toxicity.

**Table 5:** Calculated stability scores (eV.) for non-covalent binding of GaL1(SN)I and L1 at the preferred receptor site of selected cancer-mediating proteins

		Pro-cancer mediating proteins				
Compound	3EQM		RMSD (Å) of the pose	4DRH		RMSD (Å) of the pose
L1	− 6.36		1.86	− 6.99		1.15
GaL1(SN)I	− 7.46		0.757	− 8.36		1.38

### Spectroscopic titrations of GaL1-L3(SN)I with BSA displacement assays with site markers.

**Fig. 5:**
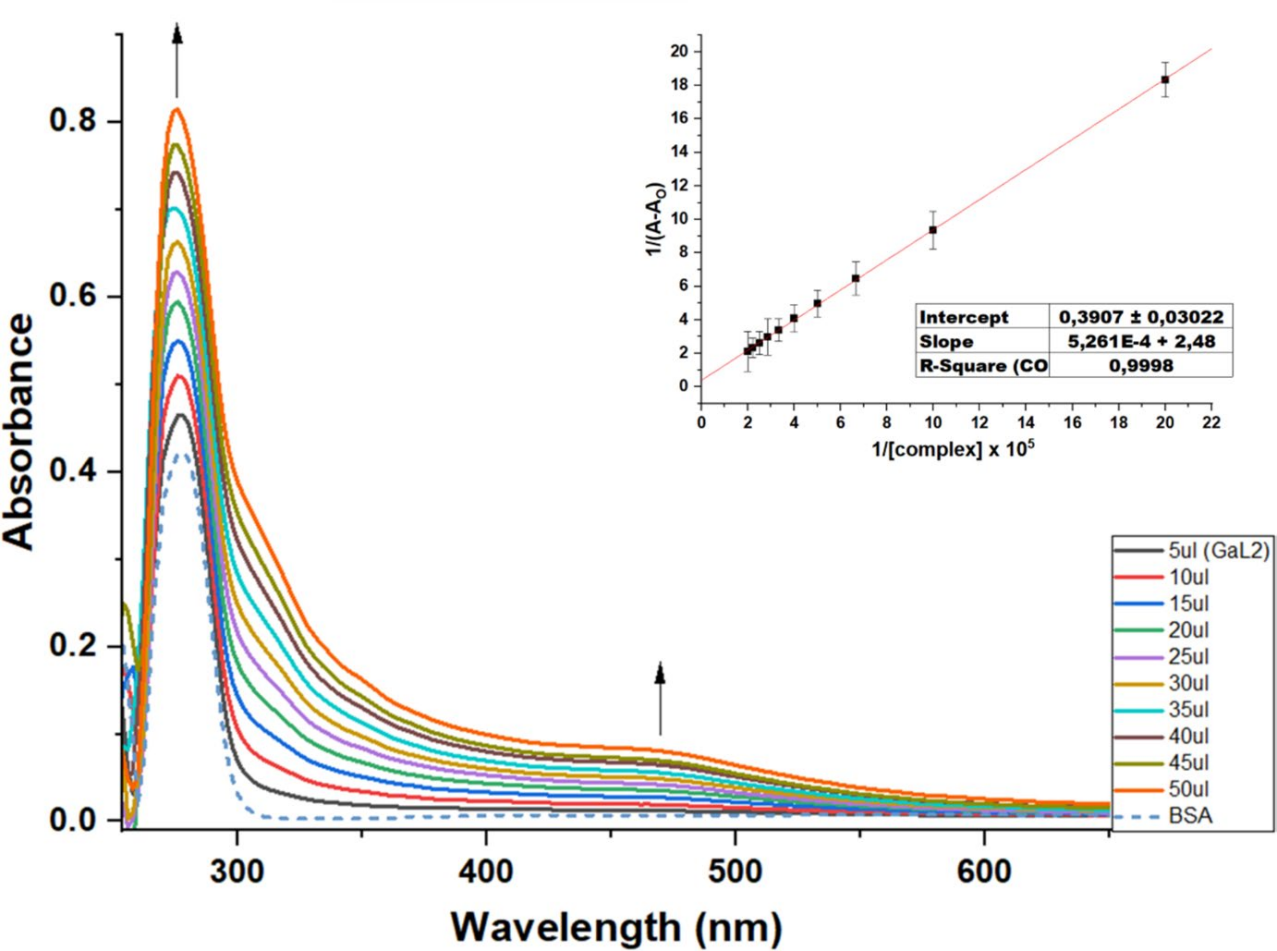
UV–Visible absorption spectra for the titration of 10 uM BSA (in PBS buffer at pH = 7.2) with GaL2(SN)I. The insets are the double reciprocal plot of $\frac{1}{({A}_{o} - A)}$ *versus* $\frac{1}{\left[\mathrm{Complex}\right]}$

Efficient binding and uptake of pharmaceutical compounds by carrier proteins such as albumins and transferrins play a pivotal role in their biodistribution and subsequent delivery to target tissues for therapeutic efficacy [68]. Bovine Serum Albumin (BSA) is homologous to Human Serum Albumin (HSA) and is commercially available and a relatively cheaper model protein for in vitro testing of the feasibility of the binding, serum‐mediated transportation, delivery, and overall pharmacokinetics in tissues. The UV–Visible absorption spectrophotometric titrations of BSA with variable amounts of complexes (GaL1-L3(SN)I) produced spectral changes depicting hyperchromic effects (48–54%) at 282 nm along with modest blue-shifts (Figs. 5 and S35). Such spectral changes are consistent with reported trends in the literature, where coordination of a metal complex induces slight unwinding of the protein’s polypeptide chains [69]. This exposes some of the buried and higher molar absorptivity microenvironments, such as those of BSA’s relatively polar tryptophan residues, thereby accounting for the observed increase in absorbance and hypsochromic shift in the wavelength of maximum absorbance (*λ*_max._). The apparent associative constants (*K*_app_) for the binding onto BSA by the complexes, GaL1-L3(SN)I, for their onward transportation and chaperoning to their site of anticancer therapy were determined by fitting the data to a double‐reciprocal plot of $\frac{1}{({A}_{o} - A)}$ *versus* $\frac{1}{\left[\mathrm{Complex}\right]}$ and were found to be 6.52, 5.26 and 4.37 × 10^4^ M⁻^1^ (Table 6). GaL1(SN)I has the highest affinity for the BSA receptors, and this is likely due to the comparatively better conformational flexibility and compact structure of its spectator ligand (L1), which allows stronger close-up contacts with the amino acid residues on the BSA. The differences in their conformational and steric features, combined with the formal positive charge of the octahedral complexes (which is higher in the dicationic GaL1(SN)I complex), facilitate the optimal hydrophobic and electrostatic contacts at the protein’s interdomain interfaces. Thus, the GaL1(SN)I-BSA adduct is the most stabilized. Furthermore, the binding of these complexes to BSA is enhanced even more by the presence of the free amino donor group on the thiosemicarbazide moiety. In all cases, it may facilitate stronger associative metallo-ligand interactions with specific BSA binding regions, particularly at thiol-containing and hydrophobic pockets enriched in methionine and cysteine residues [70, 71]. Also significant is that, in the hypoxia microenvironment, as is common for most tumor cells (where the pH is relatively lower), the moderate-to-large binding constants (hence moderate affinity for BSA) exhibited by these octahedral complexes also ensure their reversible detachment at the targeted points of therapy in the cells, thereby simultaneously improving their systematic serum transportation and delivery in cancer cells. Importantly, the strength of the molecular interactions, aqueous stability during transportation to the diseased cells, as well as the substitutional reactivity, all determine the overall pharmacokinetic profiles and hence the anticancer therapeutic effect of metallo drugs. The preferred binding site of the complexes on BSA was determined through a competitive binding assay for GaL1(SN)I (as the lead complex, which showed promising in vitro cytotoxicity) using BSA site markers ibuprofen (site I) and warfarin site (II), (Fig. S36). GaL1(SN)I and its analogues strongly preferred binding to site (II) of BSA. Analysis of the Scatchard plots indicated a single binding site per ligand (n = 1), (Fig. S37 and Table S9).

**Table 6:** Calculated binding association constants (K_*app*_) of GaL1-L3(SN)I for the preferred BSA receptors

Complex	*K*_app_, 10^4^ M^−1^	Hypercromism (%)
GaL1(SN)I	6.42 ± 0.1	54
GaL2(SN)I	5.26 ± 0.3	48
GaL3(SN)I	4.37 ± 0.4	51

### Spectroscopic titrations of GaL1-L3(SN)I with DNA-ethidium bromide (the competitive assay), and GaL1(SN)I with Hoechst-DNA.

DNA is the primary biological target for metal-based chemotherapeutics in oncology. In the cell nuclei, metallo drugs (complexes) can chemically react or non-covalently associate with the complementary groups within the DNA double helix through several distinct binding modalities, triggering structural changes that result in necrotic and/or apoptotic death of the rapidly growing cancer cells. They can form covalent bonds with nucleobases. They can also form non-covalent associative metallo-DNA pairs by inserting the planar motifs of their ligand frameworks between the DNA base pairs, resulting in stabilized π-stacked structures (intercalation). Some complexes mutually associate non-covalently with groups in the minor or major grooves (groove binding), or electrostatically with the negatively charged phosphate backbone of DNA (electrostatic association) [72, 73]. These interactions can exert a cytotoxic effect on the rapid DNA replication and transcription of malignant cells by disrupting the hydrogen-bonding network among nucleobases, thereby inducing apoptotic signalling pathways [74].

**Fig. 6:**
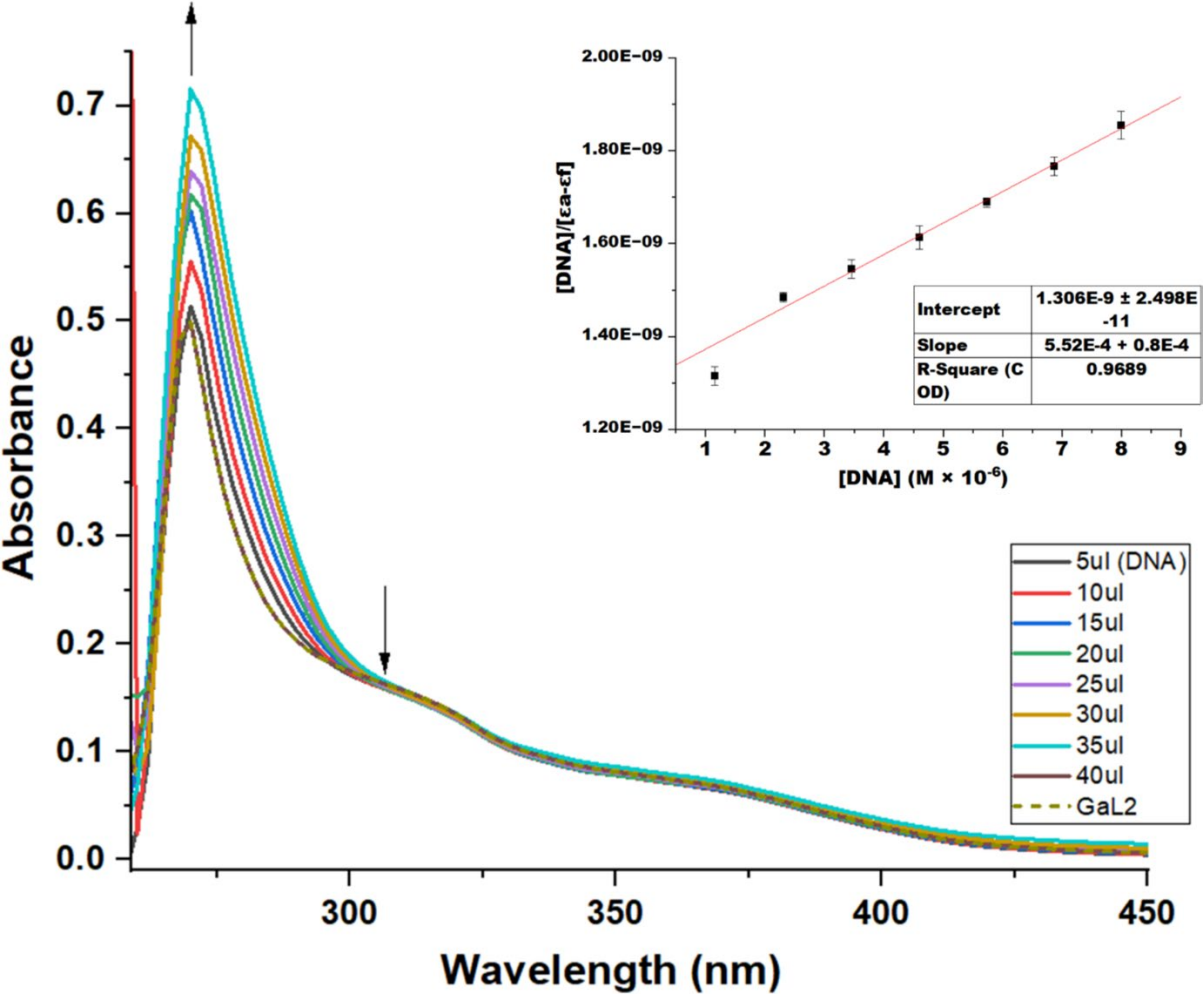
UV–Visible absorption spectra of A) GaL1(SN)I, B) GaL2(SN)I, C) GaL3(SN)I, in PBS buffer at pH = 7.2 upon addition of increasing CT-DNA concentration (0—18 μM). Inset is the linear plot of $\frac{[DNA] }{({\varepsilon}_{a} - {\varepsilon}_{f})}$ *versus* [*DNA*]

The binding affinities of GaL1-L3(SN)I towards DNA were probed by titration, and the progress monitored by UV–Visible molecular absorption spectroscopy. Incremental additions of CT-DNA to a fixed concentration of the octahedral complexes induced pronounced hyperchromic changes (22–30%) in the intensities of the band around 268 nm with distinct isosbestic points at 310 nm, (Figs. 6 and S38). The absorbance decreases occurred with minor bathochromic shifts in the *λ*_max_, which are attributed to interactions between the cationic octahedral Ga(III) complexes and groups on the DNA. The distinct isosbestic points also suggest the establishment of a non-covalent binding equilibrium between the complexes and DNA. By plotting $\frac{[\mathrm{DNA}] }{({\varepsilon}_{a} - {\varepsilon}_{f})}$ *versus* [DNA] the intrinsic binding constants (*K*_B_) (whose magnitude correlates with the affinities of the complexes for the donor groups on the CT-DNA) were estimated from the slopes and intercepts of the linear plots. The *K*_B_ values are collected in Table 7. The magnitudes of the binding constants progressively increased in the order: GaL3(SN)I < GaL2(SN)I < GaL1(SN)I. The trend of the binding (affinity constants) correlates inversely with the bulk size of the *bis*-(azaaryl)amine in the octahedral coordination sphere and the flexibility of tridentate chelates. Consequently, the complex with the bulkiest and least flexible chelate (GaL3(SN)I) exhibits the weakest binding affinity. Moreover, the trend in binding affinities of the complexes aligns with their reactivity rates. Thus, the steric properties of the *bis*-(azaaryl)amine of these complexes and their reactivity modulate the strength of the non-covalent interactions and hence their relative DNA-binding affinities. In summary, the spectral changes observed in these titration experiments allude to the existence of associative interactions (upon binding of the metal complexes) that have the potential to change the conformational structure of the double helix. The interaction may trigger the onset of cellular pathways that may terminate in apoptotic events or repair of the impaired structures in recalcitrant cancer cells [75]. Such DNA structural perturbations are due to one or a combination of the partial or full insertion of the octahedral Ga(III) complexes into the nucleobases of DNA, docking into groove binding, and electrostatic modes of intimate guest–host associative interactions [76].

**Table 7:** Binding affinity constants of GaL1-L3(SN)I for DNA

Complex	K_app_, 10^4^ M^−1^	Hyperchromism (%)
GaL1(SN)I	6.99 ± 0.5	22
GaL2(SN)I	5.52 ± 0.3	30
GaL3(SN)I	2.92 ± 0.2	28

**Fig. 7:**
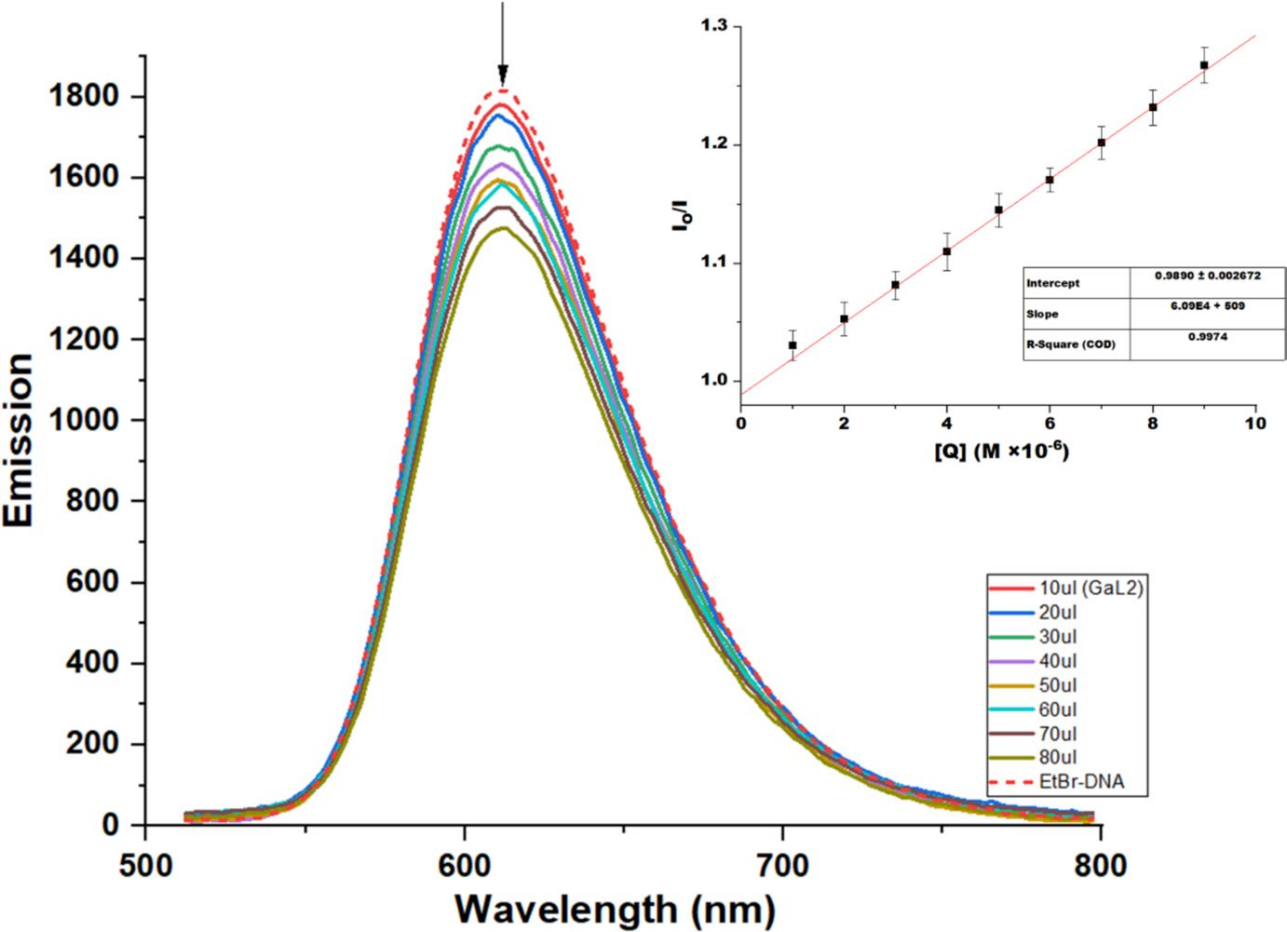
Fluorescence emission spectra depicting the quenching upon addition of A) GaL1(SN)I, B) GaL2(SN)I, and C) GaL3(SN)I, to CT-DNA-EB. The inset is the Stern–Volmer plot of $\frac{{I}_{0}}{I}$ vs. [Q], Q = GaL1-L3(SN)I complexes

DNA-ethidium bromide (EtBr) fluorescence titrations of the octahedral Ga(III) complexes (as DNA-competitive interactive displacers of EtBr and emission quencher)s were performed. The DNA-EtBr reagent is highly emissive due to the enhanced structural rigidity of the EtBr when embedded between the base pairs of DNA [77]. Consequently, fluorescence quenching of this reagent as a result of partial or complete EtBr displacement is observed, provided the incoming ligand (metallo-drug) has a planar geometry or coordinated ligand moieties that are planar and can insert into base pairs, resulting in stronger DNA-metallo-drug pairs [78]. Upon the incremental addition of the GaL1-L3(SN)I complexes to a fixed concentration of the [EtBr-DNA] adduct, a progressive decrease in fluorescence intensity (12–18% quenching) was observed for all complexes (Figs. 7 and S39, S40), indicating potential displacement of the bound EtBr [79, 80]. The indirect binding strength constant (*K*_sv_) deduced from the Stern–Volmer plots (and analysis for the GaL1-L3(SN)I-DNA non-covalent interactions) increased in the order: GaL3(SN)I < GaL2(SN)I < GaL1(SN)I with the apparent bimolecular quenching rate constants (*k*_q_) in the range of (1.25–1.94) × 10^12^ M^−1^ s^−1^ (Table 8). The displacement of ethidium bromide by the GaL1-L3(SN)I complexes was spontaneous and thermodynamically favorable, as reflected by the negative Gibbs free energy change, which ranged from − 19.11 to − 20.00 kJ mol⁻^1^. Phenanthridine-based complexes GaL2(SN)I and GaL3(SN)I have higher binding affinities (K_sv_) and *k*_q_ values compared to GaL1(SN)I. Their enhanced displacement of EtBr is attributed to the partial insertion of their planar fragments, as well as mutual and complementary non-covalent associations between the hydrophobic and planar aromatic surfaces of L2-L3 in the DNA grooves. The hydrogen bonds within the partially-inserted substructures and the van der Waals hydrophobic interactions collectively stabilized the formed non-covalent Ga(III) complex-DNA pairs. Consequently, the insertion of the planar motifs occurs at faster rates and is irreversible, as observed in the quenching of the fluorescence. The order of the binding constants alludes to the relative sizes of the planar aromatic motifs of the complexes, pointing to their partial insertion into DNA bases. In contrast, results from the displacement titrations of Hoechst-DNA by the lead cytotoxic complex GaL1(SN)I (Figs. S36—S37, and Table S9), supported by computational molecular docking simulations, classify these complexes as moderate to strong groove binders (*K*_B_ ≈ 10^3^). Collectively, these findings suggest that the GaL1-L3(SN)I complexes exhibit a bimodal DNA-binding mechanism, engaging in both groove binding and partial intercalation.

**Table 8:** Binding parameters (K_sv_, ΔG) and rate constants (*k*_q_) deduced from the Stern–Volmer plot of $\frac{{I}_{0}}{I}$ *vs.* [Q], Q = GaL1-L3(SN)I complexes

Complexes	*K*_sv_, 10^3^ M^−1^	ΔG, kJ mol^−1^	*k*_q_, 10^12^ M^−1^ s^−1^	*n*	% change in Emission Quenching
GaL1(SN)I	2.23 ± 0.5	-19.12 ± 2	1.25 ± 0.1	0.83	14
GaL2(SN)I	3.04 ± 0.5	-19.88 ± 1	1.69 ± 0.1	1.35	18
GaL3(SN)I	3.19 ± 0.3	-20.00 ± 2	1.94 ± 0.2	1.12	12

### In vitro cytotoxicity evaluation of ligands L1-L3 and their complexes GaL1-L3(SN)I against human cancer cell lines.

**Fig. 8:**
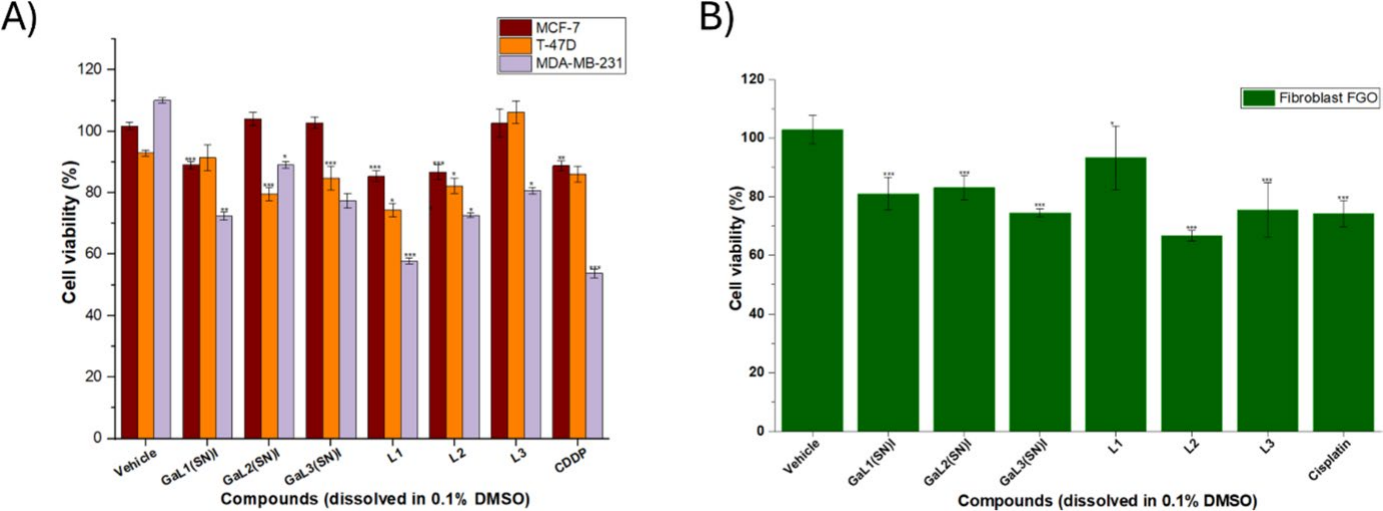
Single-dose treatments of MCF-7, T-47D and MDA-MB-231 breast (A), and (B) a non-cancerous fibroblast (FGO) cell line, with the ligands (L1-L3) and GaL1-L3(SN)I complexes. Cells were treated with vehicle (0.1% DMSO) or 10 µM of the test compounds for 48 h, followed by the MTT cell viability assay. Graphs represent the mean cell viability (± SD) of three independent experiments performed in quadruplicate. Data were analysed using the parametric unpaired t-test by GraphPad Prism 8.0 software, where *p < 0.05, **p < 0.01, ***0.001, and ****0.0001

The in vitro MTT assay is a well-established method for assessing the anticancer potential and cytotoxicity of novel compounds during the early phases of drug discovery. It allows rapid screening of compounds against various cancer and normal cell lines, facilitating the identification of selectively active candidates and the early elimination of candidates exhibiting undesirable toxicity profiles. The cytotoxic activity of the ligands **L1**-L3 and their corresponding GaL1-L3(SN)I complexes was assessed at a fixed concentration of 10 μM against a panel of breast, pancreatic, cervical cancer cell lines and a non-cancerous fibroblast (FGO) cell line. The GaL2-L3(SN)I complexes had no significant inhibitory activity against all seven tested cancer as well as the benign FGO cell lines. Only GaL1(SN)I exhibited the modest activity, reducing the viability of the triple-negative breast cancer MCF-7 cell line by 10% (Figs. 8 and S41 and the corresponding statistical data in Tables S10–S11), while the other two Ga(III) complexes’ activities were much lower. Similarly, the complexes, GaL1-L3(SN)I showed minimal cytotoxicity against T47D human breast cancer cells, reducing their cell viability by 5, 20 and 18%. Between them and their free ligands (L1-L3), only GaL1(SN)I and L1 had cytotoxicity of more than 10% against the aggressive MDA-MB-231 cell line, reducing cell viability to 76% and 58%. All metal complexes and ligands exhibited less than 5% inhibitory effect against the pancreatic cancer CFPAC-1 cell line, indicating the high intrinsic resistance of the cells towards these compounds (Fig. S41). GaL1(SN)I and L1 demonstrated promising inhibitory effects on the PANC-1 cell line, reducing cell viability by 22 and 25%. All the tested compounds were inactive against cervical CaSki cells. However, L1 and GaL1(SN)I suppressed HeLa cells’ viability by 20 and 23%. Promisingly, the inhibitory activity of L1 and GaL1(SN)I on HeLa cells is comparable to that of clinical cisplatin, indicating their potential as lead candidates for further anticancer evaluation. The generally poor cytotoxicity observed for the metal complexes can probably be ascribed to their high kinetic inertness, which limits their efficient intracellular interactions with critical biomolecular targets. The complexes also exhibited less than 20% toxicity toward the non-cancerous fibroblast (FGO) cell line (Fig. 8).

## Conclusions

Three octahedral Ga(III) complexes incorporating *bis*-(azaaryl)amine ligands with varying degrees of conformational flexibility and formal charges were synthesized, fully characterized, and evaluated for their iodide substitution reactivity with biologically relevant nucleophiles, Tu and Guan. The rate of iodide substitution was the highest for the dicationic GaL1(SN)I complex, which contains the relatively flexible L1 chelator. In contrast, the rates of substitution from the monocationic complexes bearing the more rigid proligands, L2 and L3, were lower, despite their extended π-conjugation. The complexes’ binding affinities towards BSA and DNA increased in the order: GaL3(SN)I < GaL2(SN)I < GaL1(SN)I. Competitive binding studies, complemented by molecular docking simulations, suggest that the complexes interact with DNA primarily through groove binding while also exhibiting characteristics of partial intercalation. The order in the rate of substitution, the affinities of the complexes on biological molecules (DNA/BSA), correlate with DFT predictions and molecular docking simulations. The GaL1-L3(SN)I complexes and ligands were inactive against the majority of the tested cancer cells, except for GaL1(SN)I and L1, which exhibited inhibitory effects comparable to those of clinical cisplatin against breast (MDA-MB-231) and cervical (CaSki) cell lines.

## Supplementary Information

Below is the link to the electronic supplementary material.

Supplementary file1 (DOCX 8954 KB)

## Data Availability

No datasets were generated or analysed during the current study.
