# Supplementary material for: Octahedral [(8-quinolinyl/phenanthridinyl)amine)Ga(III)] complexes, reactivity, affinity for biomolecules, anti-cancer and computational evaluations
Source: React Kinet Mech Catal. 2026 May 14;139(4):3501–31. doi: 10.1007/s11144-026-03129-6 (PMC13446110; doi:10.1007/s11144-026-03129-6)
Supplement: Supplementary file 1 [file 11144_2026_3129_MOESM1_ESM.docx]

**Supplementary Information (SI)**

**Octahedral [(8-quinolinyl/phenanthridinyl)amine) Ga(III)] complexes, reactivity, affinity for biomolecules, anti-cancer and computational evaluations.**

Phakamani C. Dlamini *^a^*, Thato T. Medupe*^c^*, Lucy W. Macharia*^b^*, Karabo Serala*^b^,* Sharon Prince*^b^*, Gregory S. Smith *^c^*, Irvin N. Booysen *^a*^*, Allen Mambanda *^a*^*

*^a^School of Agriculture and Science, University of KwaZulu-Natal, Private Bag X01, Scottsville 3209, Pietermaritzburg, South Africa.*

*^b^Department of Human Biology, University of Cape Town, Faculty of Health Science, Observatory 7925, South Africa.*

*^c^Department of Chemistry, University of Cape Town, Private Bag X3, Rondebosch 7701, South Africa.*

**S1. Materials**

The following reactants and reagents such as N-iodosuccinimide, 8-bromoquinoline, 2-bromo-2-methylaniline, trifluoracetic acid, 8-bromoquinoline, 2-formyl phenylboronic acid, 1,2-dimethoxyethane, Pd_2_(dba)_3_, sodium tert-butoxide, ammonium hexafluorophosphate, phosphate buffered saline tablets (PBS), calf thymus (CT)-DNA, Bovine Serum Albumin (BSA), guanine, thiourea and the metal precursor Ga_2_I_6_ were all procured from Sigma-Aldrich. Organic solvents were also purchased from Sigma-Aldrich and used without further purification. Organic solvents were also purchased from Sigma-Aldrich and used without further purification. All cancer cell lines were purchased from the American Type Culture Collection (ATCC, Manassas, United States of America). These included human breast MCF-7 (HTB-22), T47D (HTB-133), MDA-MB-231 (CRM-HTB-26), pancreatic PANC-1 (CRL-1469), CFPAC-1 (CRL-1918), cervical CaSki (CRL-1550), and HeLa (CRM-CCL-2) cancer cells. The key aryl halide/amine precursors for the subsequent C-N cross-coupling reactions to form the NNN ligands, viz., 4-amino-2-methylphenanthridine and 4-bromo-2-methylphenanthridine were synthesised according to the previously reported procedures.

***S2. Synthesis of precursors***

The following precursors leading to the synthesis of the phenanthridine ligand **L3**, 2-bromo-6-iodo-*p*-toluidine, 2-iodo-6-nitro-*p*-toluidine, 4-bromo-2-methylphenanthridine, **2**-methyl-4-nitrophenanthridine, and **4**-amino-2-methylphenanthridine were synthesized following a literature procedure [1]. Their characterization data is given below.

*2-bromo-6-iodo-p-toluidine*

^1^H NMR (CDCl_3_, 500 MHz): [δ ppm]: 7.45 (d, H), 7.25 (t, H), 2.21 (s, H), 5.16 (s, H). IR (cm^-1^): 3318 ν(NH), 1611 ν(C=C), 1270 ν(C-CH_3_ stretch), 549 ν(C-Br), 492 ν(C-I), 706.62 ν (aromatic C-H vibrations).

*2-iodo-6-nitro-p-toluidine*

^1^H NMR (CDCl_3_, 500 MHz): [δ ppm]: 7.97 (s, H), 7.79 (d, H), 6.50 (s, H), 2.27 (s, H). IR (cm^-1^): 3346 ν(NH), 1697 ν(C=C), 1368 ν(C-CH_3_ stretch), 1184 ν(N-O), 637 ν(C-I), 718.77 ν(aromatic C-H vibrations).

*4-bromo-2-methylphenanthridine*

^1^H NMR (CDCl_3_, 500 MHz): [δ ppm]: 8.60 (s, 2H), 8.33 (d, H), 8.08 (s, 2H), 7.90 (d, H), 7.88 (s, 2H), 7.86 (t, H), 7.73 (t, H), 2.62 (s, H). IR (cm^-1^): ν(NH), 1692 ν(C=C), 1253 ν(C-CH_3_ stretch), 1104 ν(C-N), 566 ν(C-Br), 750 ν(aromatic C-H vibrations).

*2-methyl-4-nitrophenanthridine*

^1^H NMR (CDCl_3_, 500 MHz): 9.34 (s, 2H), 8.61 (d, H), 8.54 (s, 2H), 8.11 (d, H), 7.83 (t, 2H), 7.28 (d, H); 2.70 (s, H); 1.62 (s, H). IR (cm^-1^): 3304 ν(NH), 1673 ν(C=C), 1248 ν(C-CH_3_ stretch), 1514 ν(C=N), 1115 ν(C-N), 739 ν(aromatic C-H vibrations).

*4-amino-2-methylphenanthridine*

^1^H NMR (CDCl_3_, 500 MHz): [δ ppm]: 8.57 (s, 2H), 8.02 (d, H), 7.81 (d, 2H), 7.79 (t, H), 7.65 (t, 2H), 7.28 (s, H), 6.89 (s, H), 5.01 (s, H), 2.55 (s, H). IR (cm^-1^): 3317 ν(NH), 1627 ν(C=C), 1266 ν(C-CH3 stretch), 1529 ν(C=N), 1109 ν(C-N), 755 ν(aromatic C-H vibrations).

***S3. Synthesis of N^∧^N^∧^N chelate ligands***

*N-(quinolin-8-ylmethyl)quinolin-8-amine (****L1****)*

A mass of 8-aminoquinoline (0.0649 g, 4.5 mmol) was dissolved in 25 mL of acetonitrile. While the solution was stirring, K_2_CO_3_ (0.1500 g) was added, followed by 5 mL drops of 8-(bromomethyl)quinoline (0.100 g, 4.5 mmol) and Pd(OAc)_2_ (0.0269 g, 1.2 mmol). The mixture was refluxed for 72 h at 90 °C and thereafter cooled to room temperature. The solvent was removed to near dryness under a partial vacuum, and the resulting precipitate dissolved in CHCl_3_ and backwashed with cold distilled water (3 x 15 mL). The organic phase was separated and dried over Mg_2_SO_4_, followed by purification *via* silica gel column chromatography using a 1:3 ethanol/hexane mixture. Solvent removal by rotary evaporation and further drying under vacuum resulted in an orange-brown oil. Yield: 84%.

*N-(quinolin-8-yl)-2-methylphenanthridin-4-amine (****L2****)*

Ligand, **L2,** was synthesized by reacting 8-aminoquinoline (0.2684 g, 1.3 mmol), 4-bromo-2-methyl phenanthridine (0.3519 g, 1.3 mmol), Pd(OAc)_2_ (0.0269 g, 1.2 mmol) and K_2_CO_3_ (0.12 g) in 25 mL of acetonitrile. The crude product was purified by silica gel column chromatography (1:3 EtOH/hexane), resulting in an orange-yellow precipitate which was filtered, washed with diethyl ether and dried at 30°C. Yield: 74%.

*Bis-(2-methylphenanthridin-4-yl)amine* ***(L3)***

**L3** was synthesized following the procedure of **L2** by reacting 4-amino-2-methylphenanthridine (0.0980 g, 4.7 mmol), (4-bromo-2-methyl)phenanthridine (0.1282 g, 4.7 mmol), Pd(OAc)_2_ (0.0269 g, 1.2 mmol) and K_2_CO_3_ (0.1200 g) in 25 mL acetonitrile. The crude product was purified by silica gel column chromatography (1:3 EtOH/hexane), resulting in an orange-yellow precipitate, which was filtered, washed with diethyl ether and dried at 60°C. Yield: 67%

***S4. UV-Visible absorption spectroscopy DNA binding interaction studies***

The CT-DNA stock solution was prepared in phosphate-buffered saline (PBS) at a pH of 7.2. Its purity was confirmed by a 260/280 nm UV-Visible absorption spectroscopy ratio of 1:9. DNA concentration (per nucleotide) was calculated using the molar absorption coefficient (ε260 nm = 6600 M^-1^ cm^-1^) [2]. The CT‑DNA stock solution was stored at 4 °C and used within 72 h. Stock solutions of metal complexes were prepared by dissolving the complexes in 10% DMSO and diluting with PBS to get a titrating solution of 1 mM. A fixed concentration of the metal complexes (1 mM) was titrated with increasing CT-DNA solution concentrations (0-18 µM). After each addition, the mixtures were incubated for 5 min before recording the spectra. The binding studies were conducted at 37°C to simulate the mammalian cellular environment. The DNA‑binding constants (K_b_) were obtained from the Wolfe-Shimer plot (eqn*. 1*).

$\frac{[DNA]}{(\varepsilon_{a} - \varepsilon_{b})}= \frac{[DNA]}{(\varepsilon_{b}-\varepsilon_{f})}+\frac{1/{K_{B}}}{(\varepsilon_{b}-\varepsilon_{f})}$	eqn (1).

In the above equation, [DNA] represents the concentration of CT-DNA, $\varepsilon_{a}$ is the extinction coefficient of the marked absorption band at the given DNA concentration (corresponding to $\frac{A_{obs}}{\left[ Complex \right]}$. $\varepsilon_{b}$ and $\varepsilon_{f}$ are the molar extinction coefficients of the free and fully bound [complex-DNA], respectively. $K_{b}$ is calculated using the slope-to-intercept ratio taken from the plot of $\frac{[DNA]}{(\varepsilon_{a} - \varepsilon_{f})}$ versus [DNA]. The standard Gibbs free (G) energy of metal complexes bound to DNA was calculated using the van 't Hoff equation (eqn. 2) [3].

$\Delta G=\frac{-RT}{lnK_{b}}$	(eqn. 2).

**S5. Electronic spectroscopy BSA binding interaction studies**

The BSA stock solution was prepared in PBS buffer at a pH of 7.2, and its concentration was determined spectrophotometrically using an extinction coefficient of 43824 M^-1^ cm^-1^ at 280 nm. Stock solutions of metal complexes were prepared by dissolving the metal complexes in 10% DMSO and diluting with PBS to get a titrating solution of 1 mM. A fixed BSA concentration (1×10^-5^ M) was titrated with increasing concentrations of metal complexes (0- 32 µM). The complex/DNA mixtures were incubated for 5 min before taking absorption measurements at 37°C. Equal volumes of metal complexes were added to both the sample and reference cells. To determine the apparent association constant (*k*_q_), the absorbance titrations were fitted into the following equation:

$\frac{A_{0}}{A_{0}-A}=\left( \frac{\varepsilon_{BSA}}{\varepsilon_{B}} \right)+ \frac{(\varepsilon_{BSA}/ \varepsilon_{B})}{K_{q}} \times\left( \frac{1}{[Complex]} \right)$	eqn (3).

where A_0_ and A are the absorbances of BSA in the absence and presence of metal complexes, respectively, ε_BSA_ and ε_B_ are the molar extinction coefficients of unbound BSA and the fully bound metal-BSA complex (adduct of a metal complex and BSA), respectively, *K*_q_ is the apparent association constant, and [Complex] is the concentration of a metal complex. From equation (3), in a double reciprocal plot of $\frac{1}{(Ao-A)}$ *vs* $\left( \frac{1}{[Complex]} \right),$ the apparent association constant (*K*_app_) is calculated from the ratio of the intercept to the slope [4].

**S6. Ethidium bromide displacement studies / DNA competitive binding studies**

The displacement of ethidium bromide (EB) from calf thymus DNA (CT‑DNA) by various metal complexes was investigated *via* fluorescence‑quenching assay. First, a CT‑DNA–EB adduct was formed by combining CT‑DNA and EB at equimolar concentrations (10 μM each) in phosphate-buffered saline (PBS, pH 7.4). Increasing aliquots of each metal‑complex solution (0.1 mM) were added stepwise to the CT‑DNA–EB mixture. After each addition, the sample was gently mixed and allowed to equilibrate for 5 minutes at room temperature. Fluorescence emission spectra were then recorded from 540 to 700 nm at an excitation wavelength of (λ=510 nm) to monitor EB release as evidenced by quenching of its characteristic fluorescence.

**S7. Stability of complexes in DMSO and buffer solution**

The aqueous stability of each metal complex was assessed under simulated physiological conditions (37 °C). Solutions (0.1 mM) of each complex were prepared in phosphate-buffered saline (PBS) containing 2 % DMSO (v/v) and their UV–Visible spectra were recorded hourly for 24 hours. To evaluate resistance to DMSO solvolysis, the UV–Visible absorption spectra of the complexes dissolved in anhydrous DMSO were recorded at 1 h intervals for 24 hours.

**S8**. **Computational studies**

Geometry-optimized structures of the octahedral Ga(III) complexes were computed at the Density Functional Theory (DFT) level using the B3LYP/ LANL2DZ method in the Gaussian 09 suite programs [5]. The 6-311G core-valence basis set was applied to all non-metal atoms, while the LANL2DZ basis set, which uses effective core potentials, was applied to the Ga(III) metal centre [6]. In addition, time-dependent DFT (TD‑DFT) calculations were performed in DMSO as incorporated in a Conductor Polarizable Continuum Model (C-PCM) [7], in which solvent effects on the geometry are taken into account. The calculations were carried out to obtain input-optimized molecular structures, energies of the frontier molecular orbitals, bond angles and lengths, atomic charges, and predictive electronic reactivity descriptors. The optimized structures of the octahedral Ga(III) complexes were also used as input structures for molecular docking at the receptors of CT-DNA and BSA and the cancer-associated proteins.

Molecular docking simulations were carried out using Autodock tools 1.5.7 [8]. The Autodock parameter execution files were modified by adding Ga metal parameters since the default files do not have them. Crystal structures of the receptor biomolecules relevant to the treatment of cancer, *viz.*, B‑DNA (PDB:1bna), bovine serum albumin (PDB:4f5s), were retrieved from the Protein Data Bank (The Rutgers Artificial Intelligence and Data Science (RAD) Collaboratory). Before docking, all the receptor molecules were modified by removing water molecules. In the case of BSA, the second co-crystallized strand was deleted. These receptor molecules were further altered by adding non-polar hydrogens, Gasteiger and Kollman charges. The average grid box dimensions were (38 x 42 x 72) with a spacing of 0.619 Å *for B*-DNA, (126 x 86 x 102) with a spacing of 0.753 Å for BSA, (98 x 80 x 56) with a spacing of 0.881 Å for 4DRH and (94 x 98 x 86) with a spacing of 0.708 Å for 3EQM. A generic algorithm was used as a search parameter, while the output files were expressed in a Lamarckian GA. Docked conformers of the Ga(III) complex-receptor molecule were analysed and visualized using Bovia Discovery Studio 2024.

**S9**. **Rates and mechanism of iodide substitution from the octahedral Ga(III) complexes.**

Stock solutions of the octahedral Ga(III) complexes, and the nucleophiles, thiourea (Tu) and guanine (Guan) were prepared by dissolving accurately weighed amounts of each in methanol containing 0.10 M LiI. The added LiI suppresses the spontaneous solvolysis of the iodo Ga(III) complexes [9]. Solutions of nucleophiles for monitoring the rates of iodo substitution from the Ga(III) complexes were prepared at 10 - 50‐fold excess of the complexes (0.1 mM). This ensures pseudo-first‐order kinetic conditions for the substitution reactions. Equal volumes of complexes and nucleophiles (in duplicates) were thermally equilibrated (298-313 K) in tandem cuvettes in the sample holder of a UV-Visible absorption spectrophotometer. The reaction was initiated by quickly mixing the reactants and recording the absorbance of the solution as a function of time at a set wavelength. Bimolecular rate constants, *k*_2,_ for the iodide substitution from the octahedral Ga(III) complexes were deduced from the linear plots of the observed rate constants at 298 K and the nucleophile concentration, [nucleophiles]. Observed rate constants and respective *k*_2_ values at variable temperatures (298 - 313 K) were measured using the 30-fold nucleophile. Data of *k*_2_ were plotted according to the Eyring equation from which the activation parameters (Δ𝐻^≠^ and Δ𝑆^≠^) were estimated.

**S10. Cell culture growth, maintenance and treatments**

The MDA-MB-231 triple negative breast cancer, PANC-1 pancreatic cancer and HeLa cervical cancer cell lines were cultured and maintained in Dulbecco’s Modified Eagle Medium (DMEM), the CFPAC-1 pancreatic cancer cell line in Iscove’s Modified Dulbecco’s Medium (IMDM), and the oestrogen receptor positive MCF-7 and T47D breast cancer cell lines and CaSki cervical cancer cell line in Roswell Park Memorial Institute 1640 medium (RPMI-1640). The cell culture media were obtained from Gibco (Thermo Fisher Scientific, Waltham, MA, USA). Each growth medium was supplemented with 10% heat-inactivated fetal bovine serum (FBS) and 1% penicillin-streptomycin (Pen/Strep) (Gibco, MD, USA) to support optimal cell growth and prevent microbial contamination. Cells were incubated at 37 °C in a humidified atmosphere of 5% CO₂. Culture media were replenished routinely, and cells were passaged before they reached 100% confluency. All cell lines were routinely screened for mycoplasma contamination, and only mycoplasma-free cells were used in all experiments.

***Drug Treatments.***

The uncoordinated ligands (**L1-L3**) and their respective **GaL1-L3(SN)I** metal complexes were dissolved in DMSO to achieve a stock concentration of 10 mM and stored at -4 °C for subsequent use. For cell culture treatments, the 10 mM stock solutions were diluted to final concentrations of 10 μM using the respective supplemented cell culture media. The percentage of DMSO in the 10 μM (0.1%) was used as a vehicle control, and cisplatin (CDDP) was included as a reference positive control drug.

***Spectral data***

**Figure S1**. Overlay ^1^H NMR spectra of ligand **L1** and complex **GaL1(SN)I**

**Figure S2**. Overlay ^1^H NMR spectra of ligand **L2** and complex **GaL2(SN)I**

**Figure S3**. Overlay ^1^H NMR spectra of ligand **L3** and complex **GaL3(SN)I**

**Figure S4**. Overlay ^13^C NMR spectra of ligand **L1** and complex **GaL1(SN)I**

**Figure S5**. Overlay ^13^C NMR spectra of ligand **L2** and complex **GaL2(SN)I**

**Figure S6**. Overlay ^13^C NMR spectra of ligand **L3** and complex **GaL3(SN)I**

**Figure S7**. A) Overlay IR spectra of metal complex **GaL1(SN)I** and its free-ligand **L1**

**Figure S8**. A) Overlay IR spectra of metal complex **GaL2(SN)I** and its free-ligand **L2**

**Figure S9**. A) Overlay IR spectra of metal complex **GaL3(SN)I** and its free-ligand **L3**

**Figure S10**. Mass spectrum of **GaL1(SN)I**

**Figure S11**. Mass spectrum of **GaL2(SN)I**

**Figure S12**. Mass spectrum of **GaL3(SN)I**

**Figure S13**. Elemental composition of **GaL1(SN)I**

**Figure S14**. Elemental composition of **GaL2(SN)I**

**Figure S15**. Elemental composition of **GaL3(SN)I**

**Figure S16**. Overlay UV-Visible absorption spectra of A) **GaL1(SN)I** and the free ligand **L1**, B) DFT simulated electronic absorption spectrum of **GaL1(SN)I**, C) Overlay UV-Visible absorption spectra of A) **GaL3(SN)I** and the free ligand **L3**, D) DFT simulated electronic absorption spectrum of **GaL3(SN)I**.

**Figure S17**. Overlay UV-Visible absorption spectra of A) **GaL2(SN)I** and the free ligand **L2**, B) DFT simulated spectrum of **GaL2(SN)I**.

**DFT, solvent stability, substitution kinetics and cytotoxicity data**

**Figure S18.** Time-dependent stability of A) **GaL1(SN)I** in DMSO, B) **GaL1(SN)I** in PBS: DMSO (98:2) solution, monitored by UV-Visible absorption spectrophotometry for 24 h, at 298 K

**Figure S19.** Time-dependent stability of A) **GaL2(SN)I** in DMSO, B) **GaL2(SN)I** in PBS: DMSO (98:2) solution, monitored by UV-Visible absorption spectrophotometry for 24 h, at 298 K

**Figure S20.** Time-dependent stability of A) **GaL3(SN)I** in DMSO, B) **GaL3(SN)I** in PBS: DMSO (98:2) solution, monitored by UV-Visible absorption spectrophotometry for 24 h, at 298 K.

**Table S1**. Selected wavelengths used for monitoring iodide substitution from the complexes by Guan and Tu nucleophiles.

|  | **Guan** | **Tu** |
| --- | --- | --- |
| **Complexes** | **Wavelengths, nm** | **Wavelengths, nm** |
| **GaL1(SN)I** | 274 | 297 |
| **GaL2(SN)I** | 279 | 312 |
| **GaL3(SN)I** | 310 | 321 |

**Table S2.** Average observed first-order pseudo-rate constant (*k_obs_* (s^-1^)) for the iodide substitution from **GaL1(SN)I** by guanine (Guan) and thiourea (Tu) at 298 K.

| [Guan], mM | *k*_obs_ (10^-3^ s^-1^) | [Tu], mM | *k_obs_* (10^-3^ s^-1^) |
| --- | --- | --- | --- |
| 1.00 | 0.9 | 1.00 | 10.3 |
| 2.00 | 1.7 | 2.00 | 19.6 |
| 3.00 | 2.4 | 3.00 | 29.1 |
| 4.00 | 3.4 | 4.00 | 38.3 |
| 5.00 | 4.2 | 5.00 | 47.7 |

**Table S3.** Average observed first-order pseudo-rate constant (*k_obs_* (s^-1^)) for the iodide substitution from **GaL2(SN)I** by guanine (Guan) and thiourea (Tu) at 298 K.

| [Guan], mM | k*_obs_* (10^-3^ s^-1^) | [Tu], mM | k*_obs_* (10^-3^ s^-1^) |
| --- | --- | --- | --- |
| 1.00 | 0.7 | 1.00 | 7.5 |
| 2.00 | 1.4 | 2.00 | 13.6 |
| 3.00 | 2.1 | 3.00 | 21.2 |
| 4.00 | 2.8 | 4.00 | 29.1 |
| 5.00 | 3.5 | 5.00 | 36.2 |

**Table S4.** Average observed first-order pseudo-rate constant (*k_obs_* (s^-1^)) for the iodide substitution from **GaL3(SN)I** by guanine (Guan) and thiourea (Tu) at 298 K.

| [Guan], mM | k*_obs_* (10^-3^ s^-1^) | [Tu], mM | k*_obs_* (10^-3^ s^-1^) |
| --- | --- | --- | --- |
| 1.00 | 0.6 | 1.00 | 5.9 |
| 2.00 | 1.4 | 2.00 | 11.8 |
| 3.00 | 1.9 | 3.00 | 18.5 |
| 4.00 | 2.5 | 4.00 | 24.5 |
| 5.00 | 3.2 | 5.00 | 30.7 |

**Table S5.** Temperature dependence of *k_2_* for the iodide substitution from **GaL1(SN)I** by Guan and Tu, temperature range 298-313 K, in methanol

| Guan |  | Tu |  |
| --- | --- | --- | --- |
| (1/T)/K^-1^ | ***ln(k_2_/T)*** | **(1/T)/K^-1^** | ***l*n(*k*_2_/T)** |
| 0.00335 | -2.48 | 0.00335 | -2.98 |
| 0.00330 | -2.34 | 0.00330 | -2.79 |
| 0.00325 | -2.18 | 0.00325 | -2.64 |
| 0.00319 | -2.02 | 0.00319 | -2.46 |

**Table S6.** Temperature dependence of *k_2_* for the iodide substitution from **GaL2(SN)I** by Guan and Tu, temperature range 298-313 K, in methanol

| Guan |  | Tu |  |
| --- | --- | --- | --- |
| (1/T)/K^-1^ | ***l*n(*k*_2_/T)** | **(1/T)/K^-1^** | ***l*n(*k*_2_/T)** |
| 0.00335 | -1.92 | 0.00335 | -2.68 |
| 0.00330 | -1.71 | 0.00330 | -2.51 |
| 0.00325 | -1.54 | 0.00325 | -2.38 |
| 0.00319 | -1.27 | 0.00319 | -2.25 |

**Table S7.** Temperature dependence of *k_2_* for the iodide substitution from **GaL3(SN)I** by Guan and Tu, temperature range 298-313 K, in methanol

| Guan |  | Tu |  |
| --- | --- | --- | --- |
| (1/T)/K^-1^ | ***l*n(*k*_2_/T)** | **(1/T)/K^-1^** | ***l*n(*k*_2_/T)** |
| 0.00335 | -1.48 | 0.00335 | -2.43 |
| 0.00330 | -1.23 | 0.00330 | -2.25 |
| 0.00325 | -0.96 | 0.00325 | -2.07 |
| 0.00319 | -0.72 | 0.00319 | -1.92 |

**Figure S21**. Colour-coded molecular electrostatic potential surfaces (MEPS), overlaid onto the molecular structures of **GaL1(SN)I-L3***.*

**Figure S22**. A) A kinetic trace at 274 nm for the substitution of iodide from **GaL1(SN)I** (0.3 mM) by guanine (Guan, 10-fold excess), B) Kinetic trace at 321 nm for the substitution of iodide from **GaL3(SN)I** (0.3 mM) by thiourea (Tu, at 10-fold excess) at 298 K.

**Figure S23**. Kinetic traces for the substitution of chloride from **GaL2(SN)I**, A) (0.1 mM), B (0.2 mM), C (mM), (0.4 mM ), (0.5 mM ) by Guan (10-fold excess), at 298 K

**Figure S24**. Concentration dependence plot of (*k_obs_*, s^-1^) for the iodide substitution from **GaL1-L3(SN)I** by Guan**,** *I* = 0.1 M (LiI) in methanol, T = 298 K.

**Figure S25**. Eyring plots for the iodide substitution from A) **GaL1**-**L3(SN)I** by Guan, B) **GaL1**-**L3(SN)I** by Tu, temperature range of 298 - 313 K, in methanol.

**Figure S26.** A) 3D simulated images of **GaL1(SN)I** docked into BSA subdomain IIIA, B) **GaL1(SN)I** localized non-covalent bonding to BSA’s amino acid residues, C) 2D simulated images of **GaL3(SN)I** docked into BSA subdomain IIIA, D) **GaL3(SN)I** localized non-covalent bonding to BSA’s amino acid residues.

**Figure S27**. 3D simulated images of **GaL2(SN)I** docked into BSA subdomain IIIA.

**Figure 28**. A) 3D simulated images of **GaL1(SN)I** inserted in the groove of DNA, B) Key intermolecular short contact bonds in the non-covalent interactions of **GaL1(SN)I** with the N-bases of DNA, C) 2D simulated images of **GaL3(SN)I** inserted in the groove of DNA, D) Key intermolecular short contact bonds in the non-covalent interactions of **GaL3(SN)I** with the N-bases of DNA.

**Figure S29**. 3D simulated images of **GaL2(SN)I** inserted in the groove of DNA

**Figure S30.** A) 3D simulated image of **GaL1(SN)I** docked into a preferred receptor of cytochrome P450 19A1 (PDB: 3EQM), B) 2D image of **GaL1(SN)I** bonded to the protein amino acid residues. C) 2D image of **L1** docked into a preferred receptor of cytochrome P450 19A1 (PDB: 3EQM), an enzyme that is overexpressed in breast cancer cells, D) 2D image of **L1** bonded to the protein amino acid residues.

**Figure S31**. A) 3D simulated image of **GaL1(SN)I** docked into peptidyl-prolyl isomerase receptor (PDB: 4DRH), B) 2D image of **GaL1(SN)I** bonded to the protein amino acid residues. C) 3D image of **L1** docked into peptidyl-prolyl isomerase receptor (PDB: 4DRH), an enzyme that is overexpressed in breast cancer cells, D) 2D image of **L1** bonded to the protein amino acid residues.

**Figure S32**. Superimposed poses of docked ligands (blue) and reference DFT optimized ligands (grey), obtained from docking simulations of complexes on BSA.

**Figure S33**. Superimposed poses of docked ligands (blue) and reference DFT optimized ligands (grey), obtained from docking simulations of complexes on DNA.

**Figure S34**. Superimposed poses of docked ligands (blue) and reference DFT optimized ligands (grey), obtained from docking simulations of compounds on proteins responsible for cancer antiproliferation

**Figure S35**. UV-Visible absorption spectra for the titration of 10 uM BSA (in PBS buffer at pH = 7.2) with A) **GaL1(SN)I,** B) **GaL2(SN)I** and C) **GaL3(SN)I**. The insets are the double reciprocal plot of $\frac{1}{(A_{o} - A)}$*versus* $\frac{1}{\left[ Complex \right]}$

**Figure S36.** Fluorescence emission spectra depicting the quenching upon the addition of an increasing amount of A) **GaL1(SN)I** to BSA-warfarin, B) **GaL1(SN)I** to BSA-ibuprofen and C) **GaL1(SN)I** to CT-DNA-hoechst. The inset is the Stern-Volmer plot of $\frac{I_{0}}{I}$ vs [Q].

**Figure S37.** Scatchard plots derived from the displacement titrations of A) warfarin**,** B) ibuprofen by **GaL1(SN)I** from BSA, and C) hoechst by **GaL1(SN)I** from DNA.

**Figure** **S38.** UV-Visible absorption spectra of A) **GaL1(SN)I**, B) **GaL2(SN)I**, C) **GaL3(SN)I**, in PBS buffer at pH = 7.2 upon addition of increasing CT-DNA concentration (0 - 18 μM). Inset is the linear plot of $\frac{[DNA]}{(\varepsilon_{a} - \varepsilon_{f})}$*versus* [*DNA*]*.*

**Figure 39**. Fluorescence emission spectra depicting the quenching upon addition of A) **GaL1(SN)I**, B) **GaL2(SN)I**, and C) **GaL3(SN)I,** to CT-DNA-EB. The inset is the Stern-Volmer plot of $\frac{I_{0}}{I}$ vs. [*Q*], Q = **GaL1-L3(SN)I** complexes.

**Figure S40.** Scatchard plots derived from the displacement titrations of ethidium bromide by A) **GaL1(SN)I,** B) **GaL2(SN)I,** and C) **GaL3(SN)I** from DNA.

**Table S9.** Binding and quenching rate constants for Warfarin, Ibuprofen displacement by **GaL1(SN)I** from **BSA**, and Hoechst displacement by **GaL1(SN)I** from **DNA.**

| **Binding site marker** | **K_sv_, 10^3^ M^-1^** | ***k*_q_, 10^12^ M^-1^ s^-1^** | **n** | **Emission**  **Quenching (%)** |
| --- | --- | --- | --- | --- |
| **Warfarin** | 3.77 | 7.55 | 1.15 | 20 |
| **Ibuprofen** | 9.92 | 1.98 | 1.06 | 38 |
| **Hoechst** | 5.85 | 1.17 | 1.25 | 28 |

**Figure S41.** Single-dose treatments of breast (A), and (B) pancreatic cancer cells, with the ligands **(L1-L3)** and **GaL1-L3(SN)I** complexes**.** Cells were treated with vehicle (0.1% DMSO) or 10 µM of the test compounds for 48 h, followed by the MTT cell viability assay. Graphs represent the mean cell viability (±SD) of three independent experiments performed in quadruplicate. Data were analysed using the parametric unpaired t-test by GraphPad Prism 8.0 software, where *p < 0.05,**p < 0.01, ***0.001, and ****0.0001.

**Table S10**. Statistical analysis data for evaluations for CFPAC-1, PANC-1, CaSki and HeLa cells.

| **Complex** | **CFPAC-1** | | **PANC-1** | | **CaSki** | | **HeLa** | |
| --- | --- | --- | --- | --- | --- | --- | --- | --- |
|  | **% CV ± SEM** | **P Value** | **% CV ± SEM** | **P Value** | **% CV ± SEM** | **P Value** | **% CV ± SEM** | **P Value** |
| **GaL1(Sn)I** | 94.57 ± 1.40 | 0.011187 | 76.32 ± 1.82 | <0,0001 | 101.7 ± 2.85 | 0,6469 | 75.90 ± 3.28 | 0.00101 |
| **GaL2(Sn)I** | 96.64 ± 0.96 | 0.043769 | 88.23 ± 1.58 | <0,0001 | 105.2 ± 1.72 | <0,0001 | 86.20 ± 1.89 | 0.02031 |
| **GaL3(Sn)I** | 92.71 ± 1.64 | 0.003623 | 80.60 ± 1.99 | <0,0001 | 98.30 ± 4.80 | 0,7033 | 83.00 ± 4.08 | 0.0718 |
| **L1** | 91.57 ± 1.21 | 0.000418 | 71.50 ± 1.88 | <0,0001 | 97.51 ± 2.21 | 0,1647 | 78.90 ± 3.92 | 0.00361 |
| **L2** | 96.57 ± 0.57 | 0.032939 | 83.75 ± 2.00 | <0,0001 | 96.04 ± 2.88 | 0,0035 | 80.70 ± 2.98 | 0.00406 |
| **L3** | 91.25 ± 1.50 | 0.000602 | 81.40 ± 3.43 | 0.00222 | 95.12 ± 1.64 | 0,0003 | 86.99 ± 4.89 | 0.07238 |
| **CDDP** | 71.20 ± 2.52 | <0,0001 | 28.66 ± 0.20 | <0,0001 | 44.20 ± 0.10 | <0,0001 | 77.77 ± 1.57 | 0.00144 |

**Table S11**. Statistical analysis data for the cytotoxic studies evaluations for MCF-7, T-47D, MDA-MB-231 and FG0 cells.

|  | **MCF-7** | | **T-47D** | | **MDA-MB-231** | | **FG0** | |
| --- | --- | --- | --- | --- | --- | --- | --- | --- |
| **Complex** | **% CV ± SEM** | **P Value** | **% CV ± SEM** | **P Value** | **% CV ± SEM** | **P Value** | **% CV ± SEM** | **P Value** |
| **GaL1(Sn)I** | 89.00 ± 1.23 | <0,0001 | 90.74 ± 2.68 | 0.00614 | 73.08 ± 1.02 | 0.003527 | 81.00 ± 2.49 | <0,0001 |
| **GaL2(Sn)I** | 104.1 ± 2.21 | 0.3846 | 83.21 ± 1.91 | <0,0001 | 82.56 ± 3.15 | 0.015138 | 83.08 ± 1.73 | <0,0001 |
| **GaL3(Sn)I** | 102.8 ± 1.83 | 0,9837 | 76.95 ± 1.26 | <0,0001 | 93.012 ± 5.50 | 0.160907 | 74.49 ± 0.94 | <0,0001 |
| **L1** | 85.4 ± 1.73 | <0,0001 | 78.29 ± 2.18 | <0,0001 | 63.05 ± 4.84 | <0,0001 | 93.28 ± 4.42 | 0.0875 |
| **L2** | 86.8 ± 2.36 | <0,0001 | 74.36 ± 3.26 | 0.045740 | 82.87 ± 3.71 | 0.01554 | 66.62 ± 1.05 | <0,0001 |
| **L3** | 102.7 ± 4.53 | 0.8464 | 85.29 ± 3.70 | 0.02132 | 85.57 ± 2.68 | 0.02409 | 75.37 ± 3.31 | <0,0001 |
| **CCDP** | 88.83 ± 1.58 | <0,0001 | 104.08 ± 3.09 | 0.16165 | 51.79 ± 1.86 | <0,0001 | 74.21 ± 1.85 | <0,0001 |
